# Evaluation of large language models for diagnostic impression generation from brain MRI report findings: a multicenter benchmark and reader study

**DOI:** 10.1038/s41746-026-02380-4

**Published:** 2026-01-22

**Authors:** Ming-Liang Wang, Rui-Peng Zhang, Wen-Juan Wu, Yu Lu, Xiao-Er Wei, Zheng Sun, Bao-Hui Guan, Jun-Jie Zhang, Xue Wu, Lei Zhang, Tian-Le Wang, Yue-Hua Li

**Affiliations:** 1 Department of Radiology, Shanghai Sixth People’s Hospital Affiliated to Shanghai Jiao Tong University School of Medicine, Shanghai, China; 2 Department of Radiology, Wuxi No.2 People’s Hospital, Jiangnan University Medical Center, Wuxi, China; 3 Department of Radiology, Southeast University Affiliated Nantong First People’s hospital, Nantong, China; 4 Institute for Global Health Sciences, University of California San Francisco, San Francisco, CA USA

**Keywords:** Computational biology and bioinformatics, Health care, Mathematics and computing, Medical research, Neurology, Neuroscience

## Abstract

Automatically deriving radiological diagnoses from brain MRI report findings is challenging due to high complexity and domain expertise. This study evaluated 10 large language models (LLMs) in generating diagnoses from brain MRI report findings, using 4293 reports (9973 diagnostic labels) covering 15 brain disease categories from three medical centers. DeepSeek-R1 achieved the highest performance among the evaluated models on the full dataset and across different clinical scenarios and subgroups, particularly when provided with structured report findings and clinical information. A top three differential-diagnosis prompting strategy achieved superior performance, with 97.6% patient-level accuracy versus 87.1% for single-diagnosis prompting. The diagnostic performance of six radiologists was assessed with and without DeepSeek-R1 assistance on 500 reports. Integration of DeepSeek-R1 significantly improved diagnostic accuracy (AUPRC: 0.774–0.893) and reduced reading time (from 61 to 53 s), with more pronounced benefits for junior radiologists. Our findings indicate that effective automated diagnostic impression generation in brain MRI reporting requires advanced large-scale LLMs like DeepSeek-R1. With optimized prompting and input strategies, this framework may serve as a supportive tool in drafting brain MRI reports and contribute to enhanced workflow efficiency in radiology practice.

## Introduction

Brain magnetic resonance imaging (MRI) examination plays a crucial role in brain disease diagnosis and assessment^1–4^. Compared with computed tomography (CT), MRI employs multiple sequences that demand more sophisticated interpretation skills and in-depth radiological expertise for accurate diagnosis. As the utilization of brain MRI examinations continues to rise, the workload for interpreting neuroradiologists has become increasingly burdensome^5,6^. This heavy workload may lead to delays in healthcare delivery and pose significant risks, especially for junior radiologists who may lack the experience to quickly and accurately interpret complex scans^7,8^. In clinical practice, a junior radiologist may find it relatively straightforward to identify and describe a lesion on brain MRI. The crucial challenge, however, lies in summarizing all imaging findings and applying rigorous logical reasoning required to arrive at a precise diagnosis. This higher-order cognitive capability is the primary differentiator between senior and junior radiologists.

With the rapid advancement of artificial intelligence (AI), there has been a growing focus on integrating this technology into clinical radiology practice^9–11^. Current research has demonstrated the potential of large language models (LLMs) in generating radiology impressions from imaging findings, with studies focusing on specific conditions such as lung cancer^12^, focal liver lesions^13^, and intracranial tumors^14^. Several studies have even demonstrated promising results in LLM-based radiology impression generation for whole-body imaging, utilizing various model architectures including proprietary systems (e.g., GPT-4), open-source alternatives (e.g., Qwen), or specially trained language models^15–18^. Notably, current research in this domain exhibits two fundamental limitations: (1) insufficient representation of heterogeneous disease categories in the datasets, and (2) a lack of granular, disease-level performance metrics that address key diagnostic challenges from a clinical perspective. These methodological shortcomings compromise the clinical interpretability of findings and hinder their practical application in clinical settings. To the best of our knowledge, no study has systematically evaluated the performance of multiple LLMs using diverse prompting strategies for report diagnostic impression generation from brain MRI findings, nor assessed their potential utility in clinical practice.

In this study, we systematically evaluated and compared the diagnostic performance of 10 distinct LLMs spanning diverse architectures and scales for generating diagnostic impressions from brain MRI findings. In clinical practice, radiologists often refer to clinical information to formulate a final diagnostic impression. Furthermore, structured reporting has been shown to produce more homogeneous and standardized radiology reports at the linguistic level^19,20^, which may in turn enhance the machine interpretability and improve the accuracy of AI models compared to the use of free-text inputs. Therefore, our experimental design compared four input modalities to assess their different impact on diagnostic performance: (1) original report findings (free-text) only, (2) original report findings (free-text) supplemented with clinical information, (3) structured report findings only, and (4) structured report findings combined with clinical information. Furthermore, we conducted a real-world reader study to evaluate the clinical applicability of automatic diagnostic impression generation in brain MRI reporting by LLMs.

**Fig. 1:**
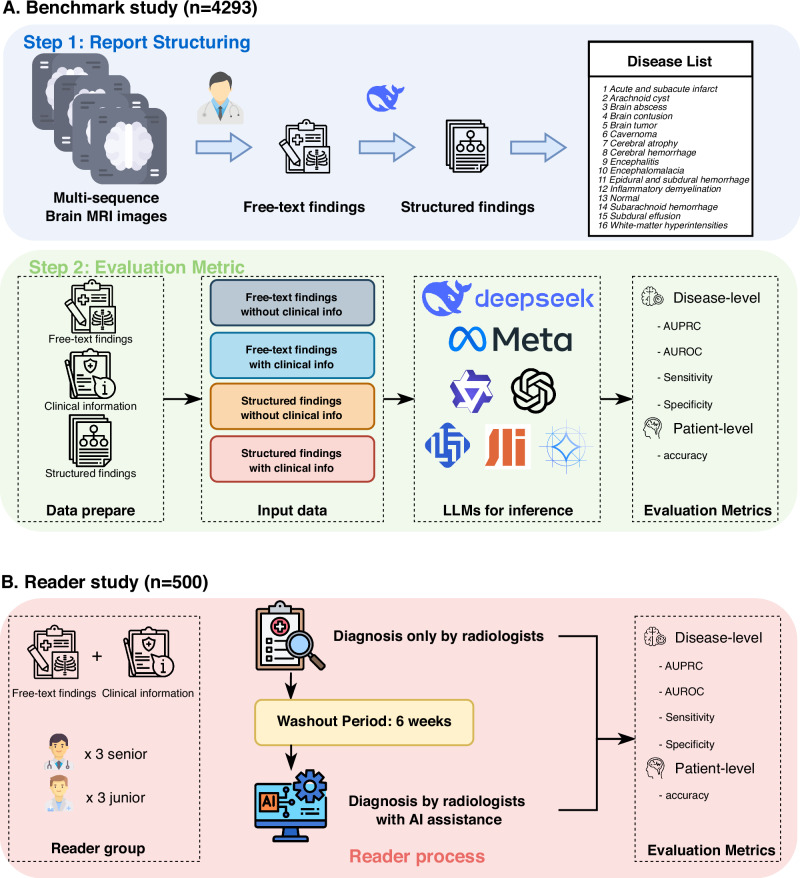
Overview of study design. **A** In benchmark study, 10 open-source LMMs were used to test for their ability to generate radiology diagnoses from brain MRI report findings in 4293 brain MRI reports, considering both structured and unstructured inputs, with and without clinical information. **B** In reader study, the diagnostic performance of interpreting radiologists was compared with and without the assistance of LLMs in 500 brain MRI reports.

The overall study protocol is illustrated in Fig. 1. Ten open-source LLMs were used to test their ability to generate radiology diagnoses from brain MRI report findings, considering both structured and unstructured inputs, with and without clinical information. The diagnostic performance of interpreting radiologists was compared with and without the assistance of LLM in the reader study. We hypothesize that LLMs are capable of generating radiology diagnoses from brain MRI report findings with a high level of clinical accuracy, even when using relatively small-scale models. The diagnostic performance is expected to improve further when structured report formats and accompanying clinical information are provided, as compared to cases without such inputs. In a real-world reader study, the integration of LLMs would not only enhance radiologists’ diagnostic performance but also shorten reading times. LLMs would hold promise as valuable decision-support tools in report drafting, especially for junior radiologists.

## Results

### Dataset characteristics

The demographic and clinical characteristics of the cohorts in the benchmark and reader studies are summarized in Table 1. The benchmark cohort included 4293 brain MRI reports with 9973 annotated neurological diagnoses from three medical centers. Notably, 10.2% (438/4293) of reports contained more than three distinct neurological diagnoses per examination. Of the included scans, 14.5% were contrast-enhanced, and 52.5% were performed in hospitalized patients, reflecting a clinically diverse population. Detailed patient demographics and prevalence distributions of all 16 neurological diagnoses across the three centers are provided in Table S1.

**Table 1:** Demographic and clinical characteristics of the whole data set

Variable	Benchmark study	Reader study
No. of report	4293	500
Age		
18–44 years	591 (13.8%)	93 (18.6%)
45–64 years	1616 (37.6%)	127 (25.4%)
≥65 years	2086 (48.6%)	280 (56.0%)
Sex		
Female	2052 (47.8%)	244 (48.8%)
Male	2241 (52.2%)	256 (51.2%)
No. of contrast-enhanced MRI	621 (14.5%)	103 (20.6%)
No. of hospitalized patients	2252 (52.5%)	277 (55.4%)
No. of diagnosis	9973	1348
Acute and subacute infarct	548 (5.5%)	68 (5.0%)
Arachnoid cyst	281 (2.8%)	30 (2.2%)
Brain abscess	39 (0.4%)	16 (1.2%)
Brain contusion	218 (2.2%)	37 (2.7%)
Brain tumor	892 (8.9%)	100 (7.4%)
Cerebral atrophy	2214 (22.2%)	333 (24.7%)
Cerebral hemorrhage	384 (3.9%)	55 (4.1%)
Cavernoma	349 (3.5%)	43 (3.2%)
Encephalitis	102 (1.0%)	32 (2.4%)
Encephalomalacia	501 (5.0%)	70 (5.2%)
Epidural and subdural hemorrhage	389 (3.9%)	51 (3.8%)
Inflammatory demyelination	63 (0.6%)	20 (1.5%)
Subarachnoid hemorrhage	268 (2.7%)	41 (3.0%)
Subdural effusion	393 (3.9%)	54 (4.0%)
White-matter hyperintensities	3032 (30.4%)	398 (29.5%)
Normal	300 (3.0%)	–

Data reported as numbers of patients, with percentages in parentheses.

### DeepSeek-R1 achieved the highest diagnostic performance among the 10 models following the neural network scaling laws

**Fig. 2:**
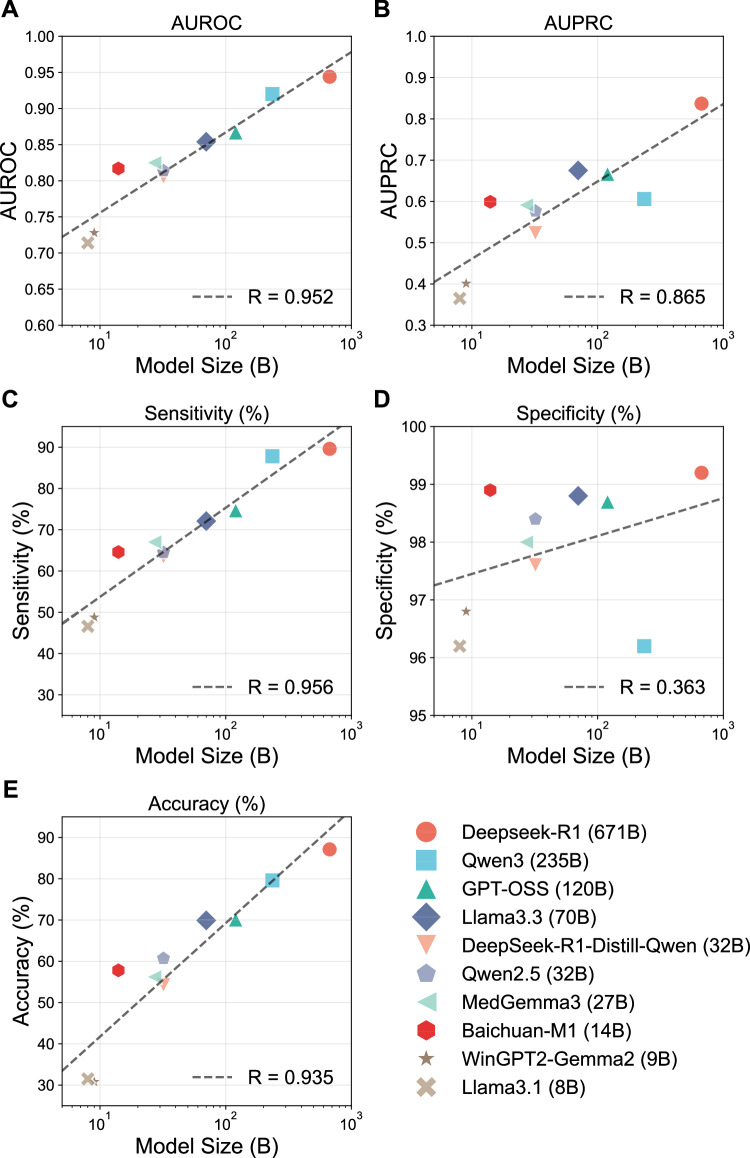
Performance metrics of 10 LLMs across different model sizes. Scatter plots were constructed to illustrate the performance of 10 LLMs in **A** AUROC; **B** AUPRC; **C** Sensitivity; **D** Specificity; **E** Patient-level accuracy.

The overall diagnostic performance of the 10 LLMs, when provided with structured findings and clinical information, is summarized in Fig. 2. DeepSeek-R1 achieved the highest performance among all models, with a sensitivity of 89.6% (95% CI: 84.8–94.3%), specificity of 99.2% (95% CI: 98.8–99.6%), patient-level accuracy of 87.1% (95% CI: 85.9–88.0%), area under the receiver operating characteristic curve (AUROC) of 0.944 (95% CI: 0.921–0.967), and area under the precision-recall curve (AUPRC) of 0.837 (95% CI: 0.779–0.894). In contrast, smaller-scale models (<10 billion parameters) exhibited markedly lower performance, achieving an AUPRC around 0.40 and patient-level accuracy of only approximately 30%, indicating limited diagnostic utility in this setting. The overall trend showed that diagnostic performance improved with increasing model size. The ranking of model performance remained consistent across the three participating centers (Fig. S1) and various strata including case complexity (Fig. S2), imaging protocol (Fig. S3) and the choice of structuring model (DeepSeek-V3 vs. Kimi-K2) (Fig. S4).

**Fig. 3:**
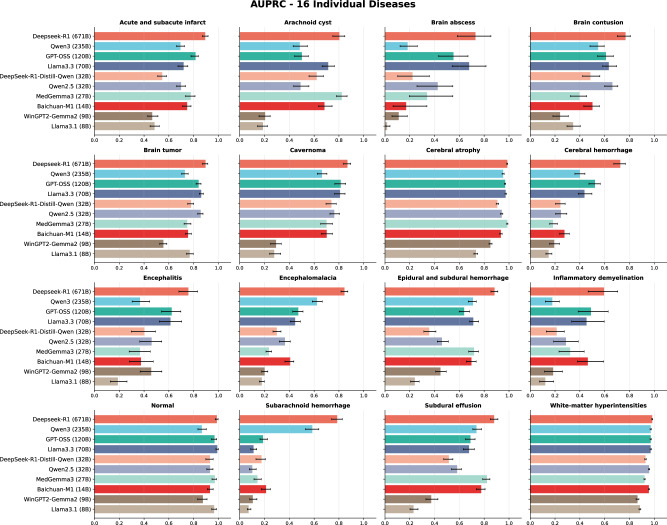
AUPRC metric of 10 LLMs for each individual disease category. Each panel shows the AUPRC (Area Under the Precision-Recall Curve) for one disease, with models ranked by performance. Higher values indicate superior performance in identifying true positive diagnoses while maintaining high precision.

For model performance comparisons across individual disease category, we prioritized the AUPRC metric due to the imbalance in disease category distribution. Compared with the other nine models, DeepSeek-R1 demonstrated superior performance in the overall diagnostic task, achieving an overall AUPRC of 0.837 (95% CI: 0.779–0.894) (Fig. 3). The per-disease AUPRC ranged from 0.595 (95% CI: 0.467–0.706) for inflammatory demyelination to 0.987 (95% CI: 0.974–0.997) for normal cases. Furthermore, DeepSeek-R1 achieved the highest performance across nearly all secondary metrics, including sensitivity, specificity, and AUROC for individual disease categories among the 10 LLMs (Figs. S5–S7).

### LLMs achieved the best diagnostic performance with structured formats and clinical information input

**Fig. 4:**
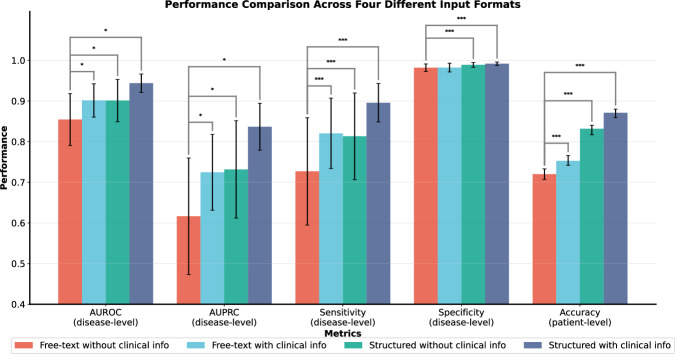
Comparison of the overall performance metrics across four different input formats for DeepSeek-R1. Our experimental design compared four input modalities to assess their different impact on diagnostic performance: (1) original report findings (free-text) only, (2) original report findings (free-text) supplemented with clinical information, (3) structured report findings only, and (4) structured report findings combined with clinical information. *<0.05, ***<0.001.

When comparing four input modalities to evaluate their impact on model diagnostic performance, DeepSeek-R1 achieved the best results when provided with structured findings combined with clinical information (Fig. 4). Compared to the free-text input format, DeepSeek-R1 using structured + clinical input demonstrated improved performance, with sensitivity increasing from 72.7% to 89.6%, specificity from 98.2% to 99.2%, patient-level accuracy from 72.0% to 87.1%, AUROC from 0.854 to 0.944, and AUPRC from 0.617 to 0.837. This trend also existed in the other nine LLMs (Table S2). With structured + clinical input setting, Qwen3 (235B), GPT-OSS (120B), and Llama3 (70B) achieved performance comparable to, and in some cases superior to, DeepSeek-R1 (671B) under the free-text input setting.

### Three-differential-diagnosis prompt outperformed single-diagnosis prompt in diagnostic performance

**Fig. 5:**
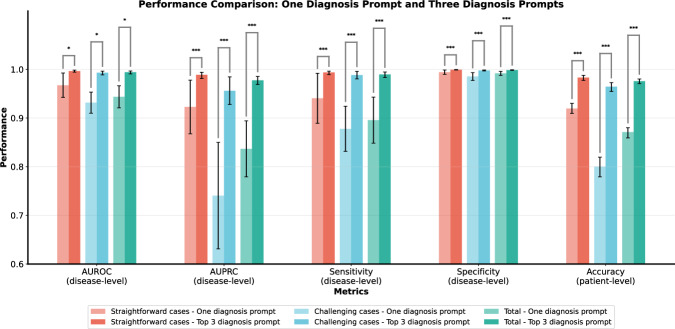
Comparison of the overall performance metrics between one diagnosis prompt and three diagnosis prompt. We compared the performances of the two prompting strategies in both straightforward cases and challenging cases, as well as across the full dataset. Accuracy values reflect patient-level complete-match. For multi-label cases, a case was scored as correct only if all reference diagnostic labels were covered, with each label appearing in the distinct top-k prediction list (*k* = 1 or 3). *<0.05, ***<0.001.

Figure 5 compares the diagnostic performance between the single-diagnosis and top-three differential-diagnosis prompting strategies. The differential-diagnosis approach demonstrated superior performance compared to the single-diagnosis method, with sensitivity rising from 89.6% to 99.0%, specificity from 99.2% to 99.9%, patient-level accuracy from 87.1% to 97.6%, AUROC from 0.944 to 0.978, and AUPRC from 0.837 to 0.994. This performance advantage was consistent across both straightforward and challenging cases, with the greatest improvement observed in the latter. Table S3 shows the detailed overall performance metrics under Top-1, Top-2, and Top-3 evaluation settings. For the full cohort, MRR increased from 0.960 (top-1) to 0.973 (top-2) and 0.975 (top-3), indicating that the correct diagnosis is typically ranked first and only rarely falls to second or third place.

### Integration of DeepSeek-R1 significantly improved diagnostic performance, especially for junior radiologists

**Fig. 6:**
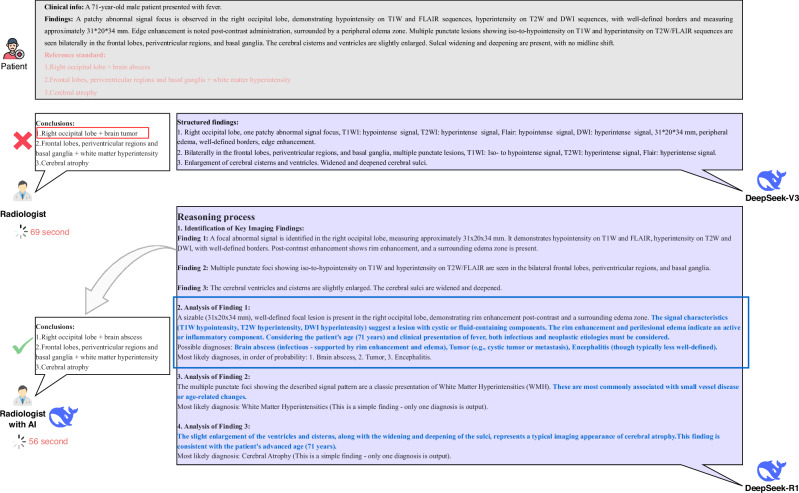
Example of report diagnosis generating process by DeepSeek-R1 in real world reader study. A junior radiologist with 2 years of experience independently interpreted brain MRI report findings and formulated the diagnoses with and without AI assistance. Initially, the radiologist interpreted the brain MRI report findings without any AI support. In this instance, the radiologist misdiagnosed a brain abscess as a brain tumor. After a 6-week washout period, the same radiologist reinterpreted the brain MRI report findings with the aid of DeepSeek’s AI diagnosis and reasoning process. With the assistance of AI, the radiologist correctly diagnosed the case as a brain abscess. The interpreting time reduced from 69 s (without AI) to 56 s (with AI). The blue words indicated the reasoning process for each disease. The red rectangle showed the wrong diagnosis by the junior radiologist without the support of AI. The blue rectangle showed the reasoning process of three differential diagnosis for the wrongly diagnosis by the junior radiologist without the support of AI.

**Fig. 7:**
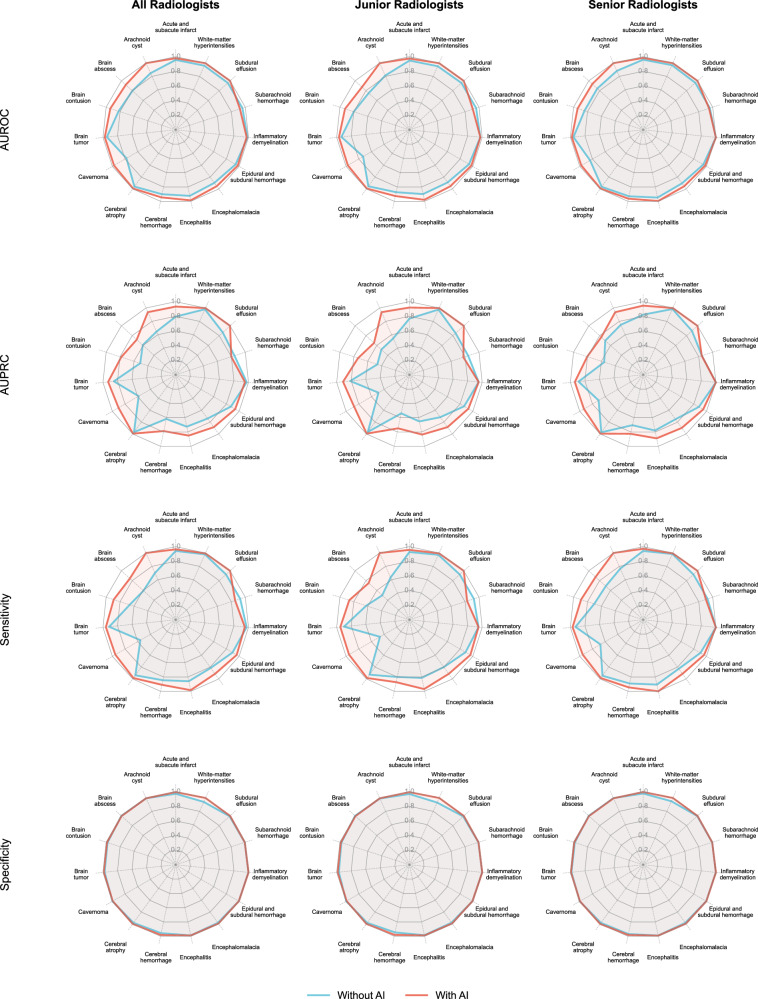
Performance metrics of unassisted radiologist and Al-assisted radiologist for each individual disease category. Radar charts were constructed to illustrate the performance of radiologists in both unassisted and AI-assisted settings across multiple metrics, including AUROC, AUPRC, sensitivity, and specificity. We repeated the analysis separately in the junior radiologists and senior radiologists.

Figure 6 illustrates a representative example of the diagnostic labels’ generation process by DeepSeek-R1 in the real-world study. Table 2 summarizes the improvement in radiologists’ diagnostic performance when they were provided with AI-generated diagnoses and corresponding reasoning processes, compared to unassisted interpretation. The integration of AI support significantly enhanced the radiologists’ ability to interpret brain disorders, increasing sensitivity by 10.7 percentage points (from 83.7% to 94.3%), specificity by 1.1 points (from 98.3% to 99.4%), and accuracy by 16.0 points (from 74.6% to 90.6%). AUROC improved from 0.910 to 0.969 (Δ = 0.059), and AUPRC from 0.774 to 0.893 (Δ = 0.119). Reading time decreased from 61 to 53 seconds. These improvements were consistent across both junior and senior radiologists, with the greatest gains observed among junior readers. Individual radiologist performance with and without AI assistance is shown in Table S4. Compared to unassisted performance across all disease categories, radiologists’ diagnostic accuracy improved with AI support in all disease categories except subarachnoid hemorrhage (SAH) (Fig. 7). This lack of improvement in SAH was primarily driven by the performance of junior radiologists.

**Table 2:** Performance of all radiologists in real-world reader study: comparison between unassisted and AI-assisted report interpretations

Parameter	Without AI	With AI	Difference	*P* value
AUROC	0.910 (0.892, 0.927)	0.969 (0.963, 0.975)	+0.059	0.002
AUPRC	0.774 (0.732, 0.815)	0.893 (0.879, 0.910)	+0.119	<0.001
Sensitivity(%)	83.7 (80.4, 86.8)	94.3 (93.3, 95.4)	+10.7	0.006
Specificity(%)	98.3 (98.0, 98.6)	99.4 (99.3, 99.5)	+1.1	0.025
Accuracy(%)	74.6 (70.2, 78.8)	90.6 (89.6, 91.7)	+16	<0.001
Reading time (s)	61 (52, 72)	53 (42, 62)	−8	<0.001

Sensitivity and specificity were compared using generalized estimating equations (GEE). The AUROC was compared using the DeLong test. Differences in the AUPRC were assessed via bootstrap-based comparison. Patient-level accuracy was evaluated using the McNemar test.

*AUROC* area under the receiver operating characteristic curve, *AUPRC* area under the precision-recall curve.

### The majority of cases modified by the radiologist–DeepSeek-R1 collaboration were corrected accurately

**Fig. 8:**
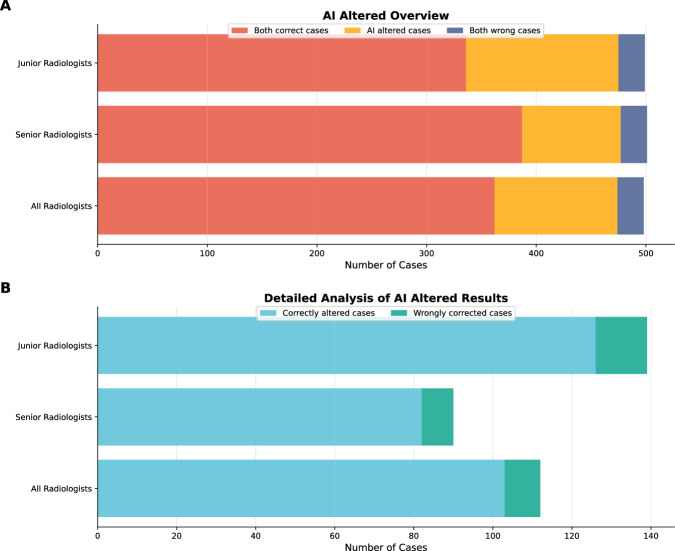
Overview of AI intervention cases by radiologist experience level. **A** Proportions of diagnostic outcomes in unassisted versus AI-assisted interpretations by radiologists, categorized as concordantly correct cases, AI-altered cases, and concordantly incorrect cases. **B** Proportion of AI-altered cases, including correctly altered cases and wrongly corrected cases among overall, junior, and senior radiologists.

Figure 8 presents the distribution of diagnostic performance and diagnostic changes in the whole radiologists. Most cases (362/500, 72.4%) were correctly diagnosed, while a small proportion of cases (10/500, 2.0%) were wrongly diagnosed by both radiologist alone and radiologist with AI support. Overall, 25.6% (128/500) of cases were altered in diagnostic impression with the support of AI. Notably, most of the altered cases (104/128, 81.25%) were correctly altered, while only a small proportion (24/128, 18.75%) of cases were wrongly altered. Junior radiologists showed a higher alteration rate (150/500, 30.0%) compared to senior radiologists (106/500, 21.2%), while most of the altered cases were correctly altered.

## Discussion

In this study, we aimed to comprehensively evaluate the diagnostic accuracy and clinical value of multiple LLMs across different input settings and prompting approaches in generating radiology report diagnostic impressions from report findings for brain MRI. Among the 10 LLMs, DeepSeek-R1 demonstrated superior performance across sensitivity, specificity, accuracy, AUROC, and AUPRC metrics, specifically when using the structured findings plus clinical information format. With one diagnosis for straightforward cases and a differential diagnosis for challenging cases prompting strategy, the integration of DeepSeek-R1 generated diagnostic impression and reasoning significantly enhanced the radiologists’ ability to interpret brain lesion descriptions and reduced interpreting time, particularly among junior radiologists.

DeepSeek-R1, a state-of-the-art open-source reasoning model with 671 billion parameters released in January 2025, has demonstrated competitive performance in third-party evaluations^21^. Previous studies have highlighted its strong performance in various medical tasks, including clinical reasoning^22^, complex diagnostic challenge^23^, and diagnostic performance in complex critical illness cases^24^. Our study further supports these findings by demonstrating that DeepSeek-R1 can effectively assist radiologists in generating radiology diagnoses from brain MRI report findings. Moreover, the reasoning ability of LLMs in radiological diagnosis improved with increasing model size, aligning with established neural network scaling laws^25^. Importantly, DeepSeek-R1 demonstrated strong performance on our task, which requires nuanced clinical inference from diverse MRI report findings. Its larger parameter scale may contribute to better handling of complex input structures, though the observed advantage likely reflects a combination of model capacity, training data, and compatibility with our structured evaluation format.

Contrary to our initial hypothesis, smaller-scale LLMs like Llama3 8b, and WinGPT2-Gemma2 9b performed poorly in generating radiology diagnoses from brain MRI report findings, with an overall disease-level accuracy of only about 30%. The performance deficit was particularly pronounced in challenging cases, where accuracy ranged from 20 to 28%, indicating limited applicability in real-world clinical settings. We speculate that generating radiology diagnoses from brain MRI findings is a task that requires deep domain-specific expertise and sophisticated interpretative reasoning especially for complex cases, which likely necessitates the use of large-scale LLMs capable of capturing such nuanced knowledge. On the other hand, small-scale LLMs face a core challenge of limited generalization in fine-grained instruction tasks due to their inherent constraints in parameter scale and data representation capability^26^. Furthermore, Baichuan-M1 (14B) and MedGemma3 (27B), two medical-specialized LLMs, demonstrated performance comparable to that of Qwen2.5 (32B), which was a larger general-purpose LLM. However, the performance was still poorer than that of DeepSeek-R1. We speculate that although domain-specialized models may possess inherent advantages for medical tasks, the task of deriving diagnostic impressions from structured brain MRI findings depends critically on model scale and general reasoning capacity, consistent with established empirical scaling laws in LLMs.

Interestingly, our study found that the LLMs had better diagnostic performance when structured report formats and accompanying clinical information were provided, as compared to cases without such inputs. Structured reporting formats, characterized by their standardized, concise, and clinically relevant information presentation, offer significant advantages in machine learning applications. These formats minimize linguistic ambiguity and data noise, thereby optimizing model learning efficiency and diagnostic specificity. In contrast, unstructured free-text formats present inherent challenges due to their variable terminology, stylistic inconsistencies, and inclusion of extraneous information, which introduce substantial noise during model training, impairing the AI system’s ability^27^. Structured reporting has been proven to improve report quality over free-text reporting in several studies^28–31^. Our previous studies also revealed the superiority of structured data input in the diagnostic performance in the application of AI^32,33^.

In addition, clinical information was found to significantly contribute to improved diagnostic performance, which was consistent with real-world clinical practice. For instance, the presence of a “history of head trauma” may guide the model toward trauma-related diagnoses such as “brain contusion,” “epidural and subdural hemorrhage,” and “subarachnoid hemorrhage.” Likewise, a documented history of “malignant tumor” raised the suspicion of “brain tumor (metastases)” and influenced the final diagnostic conclusion accordingly. A recent study also demonstrated that GPT-4V exhibited improved diagnostic performance in challenging brain MRI cases when clinical information was provided as input, compared to when it was not^34^.

In clinical practice, interpreting radiologists typically provide a definitive diagnosis for straightforward cases, while offering one or two differential diagnoses for complex or uncertain conditions to guide further evaluation and management. When the LLM generated a list of three differential diagnoses, it achieved a top-3 recall of 97.6%, higher recall than single-diagnosis (top-1) prediction, especially in clinically challenging conditions. Our study suggests a three-differential-diagnoses approach may be more appropriate for challenging cases. For challenging cases, even if the top-ranked diagnosis in the differential list is incorrect, radiologists can still refer to the full set of differential diagnoses and the accompanying reasoning process to aid in their final assessment. On the other hand, one diagnosis was sufficient for straightforward cases in clinical practice while providing three differential diagnoses was unnecessary, as this could potentially increase reading time and impose additional cognitive load on radiologists. A recent study also underscored the value of multiple differential diagnoses for complex cases in human–AI collective diagnosis task^35^. In this way, the interpreting radiologist can efficiently arrive at an accurate diagnosis, whether the case is straightforward or complex.

With a prompting strategy that provides a single diagnosis for straightforward cases and three differential diagnoses for challenging cases, the integration of AI support significantly enhanced radiologists’ ability to diagnose brain disorders, particularly among junior radiologists in the reader study. Notably, although the LLM exhibited disease-specific performance variation across the 15 diagnostic categories, its assistance helped correct radiologist errors not only in clinically challenging cases but also in seemingly straightforward ones, observed in both junior and senior radiologists. Misdiagnoses of simple cases may stem from inattention or high workload^36^, factors to which AI systems, with their consistent attention and lack of fatigue, are inherently less susceptible. Indeed, the AI-assisted model correctly diagnosed most of the straightforward cases that were missed by radiologists working without AI support. In contrast, diagnostic errors in complex cases were likely attributable to gaps in experience and knowledge^37^, which AI can partially compensate for by providing broad differential considerations. Furthermore, although the per-case time saving was modest (−8 s), this translates to an estimated 33 h of annual workload reduction per radiologist in a typical high-volume setting, thereby enhancing workflow efficiency.

It is important to note, however, that a small number of cases were incorrectly altered under AI support. Specifically, LLM assistance did not yield significant improvement in the detection of SAH among junior radiologists. This may be attributed to the high diagnostic complexity of SAH in real-world clinical scenarios, where it frequently coexists with other intracranial hemorrhages, such as intracerebral hemorrhage, cerebral contusion, or subdural hematoma. The overlapping MRI signal characteristics and morphological similarities between these entities can confound both human readers and language models. In several cases, the LLM itself misclassified SAH as subdural hematoma, likely due to shared imaging features. Given their limited experience, junior radiologists may have over-relied on the model’s output, inadvertently propagating these errors rather than correcting them. In light of the heterogeneous performance of LLMs across disease categories, radiologists should remain cautious when incorporating AI recommendations into clinical decision-making and guard against cognitive biases, such as automation bias and overconfidence in AI outputs.

Therefore, the integration of AI for the automated generation of radiology report diagnoses from report findings holds substantial clinical value, especially for junior radiologist. On one hand, it can alleviate the workload of interpreting radiologists by providing a reliable initial diagnosis, enabling more efficient review and finalization of reports. On the other hand, it serves as a valuable decision-support tool for less experienced radiologists by offering plausible differential diagnoses along with supporting reasoning process in challenging cases, thereby reducing the risk of misdiagnosis and missed diagnoses.

Our study had several limitations. First, we primarily focused on the most common brain diseases encountered in routine clinical practice. Although these conditions represented the majority of daily radiology reports, rare disorders such as inherited metabolic diseases, toxic encephalopathies, and neurodegenerative conditions were not included. Future studies should incorporate a broader spectrum of pathologies to improve the comprehensiveness of diagnostic support. Furthermore, cases with uncertain diagnoses were excluded from our analysis. The ability of LLMs to generate differential diagnoses for ambiguous brain disorders was beyond the scope of this study. Second, we grouped epidural and subdural hematomas into a single diagnostic category due to frequent lack of subtype specification in brain MRI reports. Future studies should assess diagnostic accuracy at a finer granularity to better reflect real-world interpretive challenges. Third, all radiologists interpreted the same cases in both the non-AI-aided and AI-aided phases, separated by a six-week washout period in the reader study. While this design controls for case difficulty, it may still be susceptible to subtle recall or learning effects. Fourth, the dataset was derived from a single geographic and linguistic context (Chinese radiology reports from tertiary hospitals in China). Variations in language, reporting style, and clinical practice across regions may affect model transferability. Fifth, the heterogeneity in reference standard annotation ranging from pathologic confirmation to expert opinion reflects real-world diagnostic workflows but may limit direct performance comparisons between disease groups. Sixth, our approach currently relies on radiologist-generated findings as input. This approach diverges from standard clinical practice, where radiologists formulate impressions and findings through visual interpretation of imaging data. Consequently, the generalizability of our framework to image-based diagnostic workflows remains uncertain. Although the DeepSeek-V3–based structuring pipeline showed high agreement with expert annotations in a 500-case blinded validation (98.2% complete-match accuracy at the case level), occasional errors in nuanced signal descriptions or lesion characterization may still influence downstream diagnostic reasoning. Furthermore, this study provides preliminary evidence of LLM-assisted diagnostic reasoning under controlled conditions. However, prospective trials embedded in live clinical environments, including PACS integration, usability assessment, and evaluation of effects on diagnostic confidence and patient management, are needed before any consideration of clinical deployment. Currently, automatic diagnostic impression generation directly from imaging data by LLMs has shown an unsatisfactory performance^38,39^. Given the feasibility demonstrated by our text-based approach, future vision-language models may integrate imaging analysis with our established pipeline to achieve end-to-end diagnostic support based on MR images.

In conclusion, LLMs are capable of generating radiology diagnoses from brain MRI report findings at a high level of clinical accuracy, particularly when provided with structured report formats and accompanying clinical information. With optimized prompting and input strategies, LLMs show potential to aid in drafting report impressions and improve radiologists’ workflow efficiency.

## Methods

### Datasets

This study protocol was approved by the ethics committee of Nantong First People’s hospital, the ethics committee of Wuxi No.2 People’s Hospital and the ethics committee of Shanghai Sixth People’s Hospital (approval no. 2023-KY-082 [K]) in accordance with the Declaration of Helsinki. The requirement for written informed consent was waived by the ethics committee, as this study involved a retrospective analysis of de-identified MRI reports, with no possibility of subject identification.

**Fig. 9:**
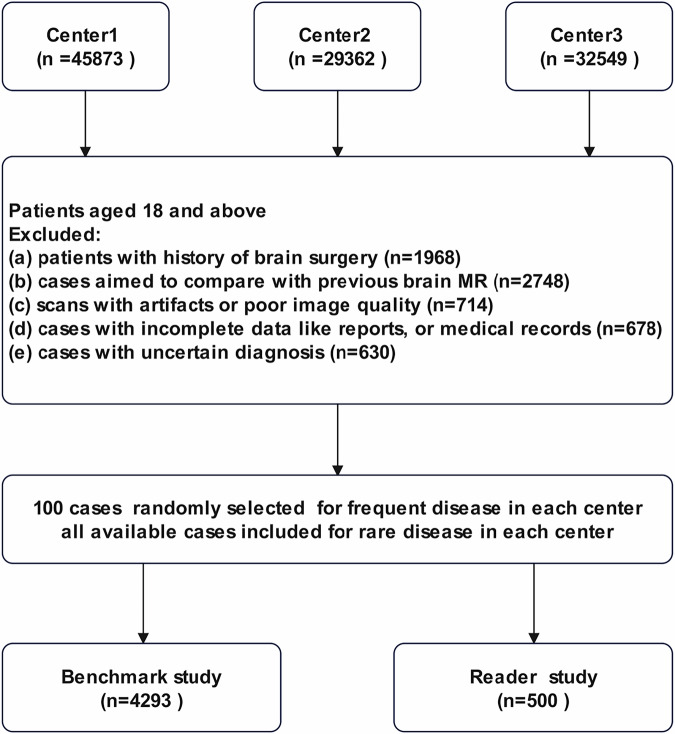
Flowchart of patient selection. Patients aged 18 and above from three centers were included after excluding those with brain surgery history, comparative scans, image artifacts, incomplete records, or uncertain diagnoses. A total of 4,293 cases were used for the benchmark study, and 500 cases for the reader study.

The data collection process is illustrated in Fig. 9. A total of 4293 brain MRI reports, along with corresponding clinical information, were retrospectively collected from three medical centers between January 2019 and December 2023. The participating centers included Shanghai Sixth People’s Hospital affiliated to Shanghai Jiao Tong University School of Medicine; Wuxi No.2 People’s Hospital, Jiangnan University Medical Center, and Southeast University Affiliated Nantong First People’s Hospital. Another 500 brain MRI reports were collected for a real-world reader study. To ensure patient privacy and data confidentiality, all radiology reports were de-identified by removing personal identifiers, replaced with unique study identification numbers. The clinical information was derived from the brief clinical history provided in the imaging order, which typically included symptoms, duration, and other key relevant context.

All included patients underwent the same MRI protocol, including T1-weighted imaging, T2- weighted imaging, T2 fluid-attenuated inversion recovery imaging, diffusion-weighted imaging for non-enhanced MRI scan, and additionally, post-contrast T1-weighted imaging for enhanced MRI scan. All brain MRI reports were originally written in Chinese, initially drafted by junior radiologists and subsequently reviewed and finalized by experienced senior neuroradiologists.

All included subjects were adult (≥18 years) and all the brain MRI reports had a confirmed diagnosis. The dataset encompassed brain MRI reports covering 15 brain disorders as well as normal cases. The 15 brain disorders included acute and subacute infarct, arachnoid cyst, brain abscess, brain contusion, brain tumor (including acoustic neuroma, meningioma, metastasis, and glioma when indicated), cerebral atrophy, cerebral hemorrhage, cavernoma, encephalitis, encephalomalacia, epidural and subdural hemorrhage, inflammatory demyelination, SAH, subdural effusion and white-matter hyperintensities (WMH). Notably, the category “white-matter hyperintensities” was almost invariably used in radiology reports to describe small vessel ischemic changes. When WMH were attributable to specific non-ischemic etiologies like inflammatory demyelination, the reports typically did not use the term “white matter hyperintensities” but instead provided a definitive diagnosis. These neurological conditions were selected based on their high prevalence in routine clinical practice, collectively representing over 95% of diagnostic cases encountered in tertiary care centers. The brain tumor was labeled as a broad category as it may be difficult to diagnose the specific brain tumor in some cases, especially in non-enhanced MRI. Case selection was stratified by diagnosis to enable a balanced evaluation of model performance across disease categories, rather than to mirror real-world prevalence. To ensure model generalizability, 100 cases were randomly selected from each diagnostic category for frequently seen diseases in each center. For rare diseases, including brain abscess, encephalitis, and inflammatory demyelination, all available cases were used due to limited sample sizes in each center.

Exclusion criteria were as follows: (a) patients with history of brain surgery; (b) cases aimed to compare with previous brain MRI; (c) scans with artifacts or poor image quality; (d) cases with incomplete data such as reports, or medical records; and (e) cases with uncertain diagnosis, whereby a definitive diagnosis could not be established after thorough discussion and review of all available medical data during the reference standard annotation.

### LLMs selection and setting

Ten distinct LLMs spanning diverse architectures and scales (8B–671B parameters) were used to generate diagnostic impressions from brain MRI findings. These LLMs included Deepseek-R1 (671B), Qwen3 (235B), GPT-OSS (120B), Llama3 (70B), DeepSeek-R-Distill-Qwen (32B), Qwen2.5 (32B), MedGemma3 (27B), Baichuan-M1 (14B), WinGPT2-Gemma2 (9B), Llama3 (8B). The panel includes mixture-of-experts (MoE) models (e.g., Deepseek-R1, 671B, GPT-OSS, 120B), dense models (e.g., Llama3-8B/70B, Qwen2.5-32B), and distilled variants (DeepSeek-R-Distill-Qwen, 32B). All support long-context inputs (≥32 K tokens), which is essential for processing full-length radiology reports. By including models with varying parameter counts, sparsity mechanisms, and computational demands, this benchmark assesses not only diagnostic accuracy but also generalization across model families and practical considerations for real-world integration in radiology workflows.

Model inference was conducted using the vLLM framework within Docker containers (v0.8.4; GPT-OSS executed with v0.10.1-oss). All experiments were run on a high-performance cluster equipped with eight NVIDIA H20 GPUs (141 GB memory each). Model architecture (MoE vs dense), scale, routing/activated capacity (for MoE), default weight/precision format, and decoding hyperparameters (temperature, top-p) are summarized in Table S5. Unless otherwise specified, KV cache was enabled and the maximum generation length was fixed at 4,096 output tokens, with decoding terminated upon reaching this limit or emitting the end-of-sequence token. We further report model-side wall-clock time per case as (a) input preparation/structuring and (b) LLM generation, with (a + b) as the model-side total, stratified by input modality, prompting strategy, case difficulty, and scan type (Table S6). Physician review time (c) was measured in the reader study and is reported in Table 2 (“Reading time (s)”).

### Practical deployment

Although our benchmarking was conducted on a research-grade GPU cluster, real-world deployment can be lightweight and scalable. For resource-constrained sites, cloud inference on de-identified, text-only findings offers elastic capacity for high concurrency with minimal on-premises hardware. In contrast, aggressive quantization/model downsizing or serving on smaller GPUs (e.g., L4/RTX-class) may reduce compute requirements but can degrade impression quality and should therefore be adopted only after local validation. To provide a practical reference, we conducted a timed simulation test using DeepSeek’s official online API service under our study prompts and structuring workflow. We observed a time-to-first-token (TTFT) of ~0.6 s for the findings-structuring step using DeepSeek-V3, while diagnostic impression generation using DeepSeek-R1 yielded TTFTs of ~0.8 s, with sustained output throughput of approximately 25–30 tokens/s. These latency measurements depend on prompt length, concurrency, network conditions, and server load. Notably, smaller distilled models typically run faster under the same serving setup.

### Radiology reports structuring and reference standard annotation

We employed DeepSeek-V3 to structure the original brain MRI reports by automatically identifying and matching radiology-specific terms using a zero-shot prompting strategy. Specifically, the “Findings” section was refined to generate concise summaries that align with each possible impression, while adhering to a standardized reporting framework. This framework adhered to fixed reporting criteria, including lesion anatomical location (e.g., frontal lobe, basal ganglia), number (e.g., one, multiple), morphology (shape, and margins such as “ill-defined oval lesion”), signal characteristics on T1-weighted, T2-weighted, FLAIR, and DWI sequences (e.g., “T1 hypointense, T2 hyperintense”), perilesional changes, and other characteristic imaging features. A representative example of the original report and its corresponding structured output was provided in Table S7. Furthermore, the “Impression” section was systematically organized to align with 16 predefined brain diagnoses, formatted as [Location] + [Disease] (e.g., “Left frontal lobe+ acute infarct”). This structured output served as the foundation for the reference standard annotation. The detailed prompt design methodology was provided in the GitHub repository at https://github.com/FrankZhangRp/BrainMIND and Google Drive https://drive.google.com/file/d/1hsjFtQLql73tYyfyaK3cKiOAbUOYIqDA/view?usp=sharing.

To quantify the fidelity of the structured findings generated by DeepSeek-V3, we conducted a blinded validation study on a randomly selected subset of 500 brain MRI reports. Two board-certified neuroradiologists, blinded to the LLM outputs, independently extracted structured labels from the original free-text “Findings” sections using the same schema as DeepSeek-V3 (anatomical location, lesion number, morphology, signal characteristics, perilesional changes, and other characteristic imaging features). Discrepancies were adjudicated by a third senior neuroradiologist to establish a consensus reference standard. DeepSeek-V3–generated structured outputs were then compared against this reference at both the case and field levels. At the case level, a case was considered correct only if all structured fields matched the reference; under this complete-match criterion, 491 of 500 cases were perfectly structured, corresponding to an accuracy of 98.2%. At the field level, accuracies were 99.6% for anatomical location, 100.0% for lesion number, 99.6% for morphology, 98.8% for signal characteristics, 99.8% for perilesional changes, and 100.0% for other characteristic imaging features. The distribution of structuring errors across fields and diagnostic categories is provided in Table S8.

In addition, we performed a predefined sensitivity analysis to evaluate whether the choice of structuring model could bias downstream benchmark results. We used Kimi-K2 (Moonshot AI), an open-source LLM from an independent development team that was not included among the 10 LLMs evaluated in the benchmark, to restructure the original free-text “Findings” using the same prompts and schema. Under the “structured findings + clinical information” setting, all 10 LLMs were re-evaluated with Kimi-K2–structured inputs. Disease-level and model-level metrics were compared between DeepSeek-V3– and Kimi-K2–structured inputs using paired t-tests with false discovery rate (FDR) and Bonferroni correction for multiple testing. The full results are represented in Table S9 and showed limited, bidirectional differences without a consistent systematic advantage for either structuring approach.

Expert-adjudicated diagnostic labels derived from brain MRI report impressions were used as reference standard annotation. In detail, structured report impressions ([Location] + [Disease]) were used as the basis for reference standard annotation and were subsequently reviewed and refined by two board-certified neuroradiologists (M.L.W. and X.E.W. with 10 and 15 years of experience). Each neuroradiologist was assigned half of the brain MRI reports for evaluation and had full access to all relevant medical records, including medical history and surgical, laboratory, pathology, and radiology records. Definitive diagnoses were either histopathologically confirmed or established through comprehensive review and consensus discussion of all relevant clinical and imaging information. Notably, a single patient could be assigned to multiple disease labels. Cases with diagnostic uncertainty or ambiguity were resolved through consensus review. If a definitive diagnosis could not be determined after thorough discussion and review, the case was classified as “uncertain diagnosis” and excluded from further analysis.

### Radiology report diagnostic accuracy assessment

The input configuration comprised four distinct modalities: (1) original report findings (free-text) only, (2) original report findings (free-text) supplemented with clinical information, (3) structured report findings only, and (4) structured report findings combined with clinical information. For each configuration, models were required to generate both a primary diagnosis and output the corresponding reasoning process. We evaluated each model’s overall performance in generating accurate radiology diagnosis impression from brain MRI report findings across all four input configurations. In addition, we conducted a disease-specific diagnostic accuracy analysis for individual clinical categories.

Since the models generated only one diagnosis by one single diagnosis prompt, this may pose a challenge in complex cases. To address this limitation, we expanded the task to require models to output three most likely differential diagnoses with supporting reasoning processes. We adopt a strict patient-level complete-match criterion for accuracy evaluation. A prediction is correct only if the set of AI-generated diagnostic labels exactly matches the reference standard label set. For multi-label cases, the model generated N ranked differential-diagnosis lists (where N was the predicted number of distinct pathologies). A case was scored as correct only if all reference diagnostic labels were covered, with each label appearing in the distinct top-k prediction list (*k* = 1 or 3). All reported top-1 and top-3 accuracies were computed under this patient-level, complete-match definition.

We then compared the performances of the two prompting strategies in both straightforward cases and challenging cases, as well as across the full dataset. Challenging cases were defined as those involving brain contusion, brain tumor, brain abscess, cerebral hemorrhage, encephalitis, and inflammatory demyelination, all of which were suggested to be difficult to diagnose, especially for junior radiologist. All other cases were categorized as straightforward cases. To better characterize whether the model identifies the most likely diagnosis, Mean Reciprocal Rank (MRR) for the ranked differential list was calculated. The detailed prompt design is available in the GitHub repository at https://github.com/FrankZhangRp/BrainMIND.

To guarantee the robustness of the findings, we conducted several multiple sensitivity analyses: (1) stratified by individual center (*n* = 3); (2) stratified by case difficulty (straightforward vs. challenging cases); (3) stratified by imaging protocol (contrast-enhanced vs. non-contrast-enhanced MRI); and (4) using structured imaging findings generated by different LLMs (DeepSeek-V3 vs. Kimi-K2).

### Real-world reader study

A subset of 500 brain MRI reports, encompassing all diagnostic categories, was randomly selected for a two-phase reader study. Two groups of radiologists with varying expertise participated in the reader study: three junior radiologists (2 years’ experience, 3 years’ experience and 4 years’ experience), and three senior radiologists (9 years’ experience, 11 years’ experience and 12 years’ experience). To minimize possible memory bias, none of the participating reviewers participated in the initial case selection. All readers were aware of the study design but blinded to the actual diagnoses. Prior to the formal assessment, they each underwent training on a set of 10 cases (which were excluded from the study) to familiarize themselves with the evaluation process.

In the first session, readers independently interpreted brain MRI report findings and formulated the diagnoses without AI assistance. In the second session, readers reinterpreted the same cases by additionally referring to AI-generated report diagnostic impression and reasoning processes after a 6-week washout period. The test cases were presented in randomized sequence to minimize bias in each session. And the reading times were recorded.

DeepSeek-R1 was used in the reader study and was provided with both clinical information and structured-format MRI report findings. Of note, we designed an adaptive prompting strategy for the reader study to maximize the effectiveness of LLM in clinical practice. Under this approach, the model was instructed to generate a single diagnosis for straightforward cases and to provide three differential diagnoses for challenging cases. Challenging cases were defined as those potentially involving brain contusion, cerebral hemorrhage, brain tumor, brain abscess, encephalitis, or inflammatory demyelination; all other cases were classified as straightforward. The detailed prompt design was available in the GitHub repository at https://github.com/FrankZhangRp/BrainMIND. The overall and disease-specific diagnostic performance of the radiologists was then evaluated with and without the support of DeepSeek-R1.

### Statistical analysis

Model performance was assessed using sensitivity, specificity, patient-level accuracy, AUROC, and AUPRC. All metrics were reported as macro-averages across disease categories, with 95% confidence intervals (CIs) estimated by cluster-based bootstrap resampling at the patient level (2000 replicates unless otherwise specified).

Comparisons between models were conducted as follows. Sensitivity, specificity, and patient-level accuracy were compared using McNemar’s test for paired proportions. AUROC values were compared using the DeLong test for correlated receiver operating characteristic curves. AUPRC values were compared using patient-cluster bootstrap and permutation-based tests. Differences in macro-averaged metrics between baseline and comparison models were further estimated with bootstrap 95% CIs, and *p* values were obtained from permutation tests with plus-one correction.

For the reader study, paired comparisons of sensitivity and specificity across cases were performed using generalized estimating equations (GEE) to account for within-reader correlation. AUROC values were compared using the DeLong test, AUPRC using bootstrap resampling, and patient-level accuracy using McNemar’s test. Reading time differences between with-AI and without-AI conditions were analyzed using linear mixed-effects models with random intercepts for readers. Per-case diagnostic correctness (correct vs incorrect) at the reader–case–condition level was analyzed using GEE with a logit link and clustering by reader to account for within-reader correlation; odds ratios and 95% confidence intervals for AI assistance are reported in Table S10.

Additional subgroup analyses were conducted for difficult versus non-difficult cases and for scan type (non-contrast vs. contrast-enhanced), with performance differences assessed using the same bootstrap and permutation framework. For disease-level subgroup comparisons (including analyses of input configurations and top-1 vs. top-3 prompting), sets of per-disease tests for each metric and setting were treated as families of related hypotheses and adjusted for multiple comparisons using the FDR procedure; the corresponding effect sizes, confidence intervals, and numbers of diseases with FDR-adjusted *p* < 0.05 are summarized in Tables S11 and S12. For comparisons of top-1 versus top-3 model outputs, case-level correctness was defined as whether all true positive and negative labels for a patient were simultaneously predicted without error.

All tests were two-sided, and *p* values < 0.05 were considered statistically significant. Confidence intervals are reported as 95% CIs unless otherwise specified. Analyses were performed in Python (v3.11), using NumPy (v1.26.4), SciPy (v1.16.1), pandas (v2.2.3), scikit-learn (v1.2.2), and statsmodels (v0.14.5), with visualization conducted using Matplotlib (v3.10.3) and Seaborn (v0.12.2). Random seeds were fixed across bootstrap, permutation, and model evaluation procedures to ensure reproducibility.

## Supplementary information

Supplementary Information

## Data Availability

The source code for model deployment, inference scripts, trained model weights and allprompts used in this work have been made publicly available at https://github.com/FrankZhangRp/BrainMIND.git and https://drive.google.com/file/d/1hsjFtQLql73tYyfyaK3cKiOAbUOYIqDA/view?usp=sharing.

## Code availability

The source code for model deployment, inference scripts, trained model weights and all prompts used in this work have been made publicly available at https://github.com/FrankZhangRp/BrainMIND and https://drive.google.com/file/d/1hsjFtQLql73tYyfyaK3cKiOAbUOYIqDA/view?usp=sharing.
