# Supplementary material for: Evaluation of large language models for diagnostic impression generation from brain MRI report findings: a multicenter benchmark and reader study
Source: NPJ Digit Med. 2026 Jan 22;9:187. doi: 10.1038/s41746-026-02380-4 (PMC12929788; doi:10.1038/s41746-026-02380-4)
Supplement: Supplementary file 1 — Supplementary Information [file 41746_2026_2380_MOESM1_ESM.pdf]

Table S1. Demographic and clinical characteristics of the three centers

Variable	Center 1	Center 2	Center 3
No. of report	1488	1405	1400
Age			
18-44y	251 (16.9%)	183 (13.0%)	157 (11.2%)
45-64y	584 (39.2%)	522 (37.2%)	510 (36.4%)
≥65y	653 (43.9%)	700 (49.8%)	733 (52.4%)
Sex			
Female	755 (50.7%)	615 (43.8%)	682 (48.7%)
Male	733 (49.3%)	790 (56.2%)	718 (51.3%)
No. of contrast-enhanced MRI	165 (11.1%)	226 (16.1%)	230 (16.4%)
No. of hospitalized patient	781 (52.5%)	756 (53.8%)	715 (51.1%)
No. of disease	3551	2848	3574
Acute and subacute infarct	208 (5.9%)	144 (5.1%)	196 (5.5%)
Arachnoid cyst	68 (1.9%)	108 (3.8%)	105 (2.9%)
Brain abscess	20 (0.6%)	4 (0.1%)	15 (0.4%)
Brain contusion	67 (1.9%)	110 (3.9%)	41 (1.1%)
Brain tumor	339 (9.5%)	303 (10.6%)	250 (7.0%)
Cerebral atrophy	879 (24.8%)	395 (13.9%)	940 (26.3%)
Cerebral hemorrhage	104 (2.9%)	117 (4.1%)	163 (4.6%)
Cavernoma	139 (3.9%)	101 (3.5%)	109 (3.0%)
Encephalitis	34 (1.0%)	37 (1.3%)	31 (0.9%)
Encephalomalacia	145 (4.1%)	162 (5.7%)	194 (5.4%)
Epidural and subdural hemorrhage	153 (4.3%)	145 (5.1%)	91 (2.5%)
Inflammatory demyelination	21 (0.6%)	22 (0.8%)	20 (0.6%)
Subarachnoid hemorrhage	70 (2.0%)	122 (4.3%)	76 (2.1%)
Subdural effusion	119 (3.4%)	125 (4.4%)	149 (4.2%)
White-matter hyperintensities	1085 (30.6%)	853 (30.0%)	1094 (30.6%)
Normal	100 (2.8%)	100 (3.5%)	100 (2.8%)

Data reported as numbers of patients, with percentages in parentheses.

Table S2. Performance metrics of 10 LLMs across four input formats

Input	Models	Size	AUROC (disease-level)	AUPRC (disease-level)	Sensitivity(%) (disease-level)	Specificity(%) (disease-level)	Accuracy(%) (patient-level)
Free-text findings	Deepseek-R1	671B	0.854 (0.791-0.918)	0.616 (0.473-0.760)	72.7 (59.5-85.9)	98.2 (97.3-99.1)	72.0 (70.7-73.3)
	Qwen3	235B	0.834 (0.769-0.899)	0.485 (0.331-0.639)	71.9 (58.0-85.7)	95.0 (93.1-96.8)	64.0 (62.7-65.5)
	GPT-OSS	120B	0.763 (0.677-0.848)	0.457 (0.292-0.621)	54.7 (37.0-72.5)	97.8 (96.4-99.1)	56.6 (55.3-58.0)
	Llama3	70B	0.719 (0.628-0.810)	0.420 (0.246-0.594)	46.0 (26.9-65.1)	97.9 (96.2-99.5)	51.6 (50.3-53.2)
	DeepSeek-R-Distill-Qwen	32B	0.711 (0.625-0.797)	0.371 (0.206-0.536)	45.2 (27.1-63.4)	97.0 (95.3-98.6)	41.3 (40.0-42.5)
	Qwen2.5	32B	0.710 (0.617-0.802)	0.378 (0.208-0.547)	44.1 (24.6-63.7)	97.8 (96.5-99.0)	45.7 (44.3-47.0)
	MedGemma3	27B	0.728 (0.636-0.820)	0.417 (0.230-0.603)	48.5 (29.8-67.2)	97.0 (94.3-99.7)	42.7 (41.1-44.3)
	Baichuan-M1	14B	0.727 (0.638-0.815)	0.424 (0.253-0.596)	47.2 (28.8-65.6)	98.2 (97.1-99.3)	43.7 (42.3-45.0)
	WinGPT2-Gemma2	9B	0.659 (0.586-0.732)	0.310 (0.152-0.468)	35.5 (19.8-51.3)	96.3 (93.7-98.8)	26.3 (24.9-27.5)
	Llama3	8B	0.643 (0.565-0.720)	0.289 (0.127-0.452)	32.9 (16.8-49.0)	95.6 (92.7-98.6)	26.6 (25.2-27.7)
Free-text findings + clinical information	Deepseek-R1	671B	0.901 (0.861-0.942)	0.724 (0.631-0.818)	82.1 (73.4-90.7)	98.2 (97.2-99.3)	75.3 (74.2-76.6)

	Qwen3	235B	0.887 (0.846-0.928)	0.549 (0.424-0.673)	82.3 (73.6-91.1)	95.1 (93.4-96.9)	68.7 (67.6-70.1)
	GPT-OSS	120B	0.810 (0.735-0.885)	0.554 (0.411-0.696)	63.9 (48.5-79.3)	98.2 (97.1-99.3)	59.4 (57.8-60.6)
	Llama3	70B	0.795 (0.717-0.873)	0.561 (0.412-0.711)	60.7 (44.4-76.9)	98.3 (97.1-99.6)	58.3 (56.9-59.8)
	DeepSeek-R-Distill-Qwen	32B	0.758 (0.688-0.827)	0.443 (0.301-0.586)	54.5 (39.9-69.1)	97.1 (95.4-98.8)	44.4 (43.0-45.9)
	Qwen2.5	32B	0.764 (0.685-0.844)	0.473 (0.324-0.622)	55.0 (38.3-71.8)	97.8 (96.4-99.2)	50.7 (49.0-52.4)
	MedGemma3	27B	0.779 (0.699-0.859)	0.502 (0.336-0.669)	58.2 (42.1-74.3)	97.6 (95.1-100.0)	47.2 (45.7-48.4)
	Baichuan-M1	14B	0.774 (0.703-0.846)	0.509 (0.365-0.652)	56.6 (41.8-71.5)	98.3 (97.1-99.4)	47.8 (46.4-49.3)
	WinGPT2-Gemma2	9B	0.684 (0.620-0.749)	0.341 (0.191-0.491)	40.6 (26.3-54.9)	96.3 (93.6-99.0)	27.2 (25.8-28.5)
	Llama3	8B	0.677 (0.604-0.750)	0.320 (0.160-0.480)	39.8 (24.9-54.7)	95.7 (93.2-98.2)	28.2 (26.9-29.6)
Structured findings	Deepseek-R1	671B	0.901 (0.849-0.953)	0.732 (0.612-0.852)	81.3 (70.7-92.0)	98.9 (98.3-99.5)	83.2 (81.7-84.1)
	Qwen3	235B	0.870 (0.809-0.931)	0.553 (0.398-0.708)	77.6 (65.1-90.2)	96.4 (95.5-97.3)	75.4 (74.3-76.6)
	GPT-OSS	120B	0.824 (0.748-0.901)	0.576 (0.424-0.728)	66.6 (50.7-82.4)	98.3 (97.3-99.4)	68.1 (66.6-69.5)
	Llama3	70B	0.781 (0.692-0.870)	0.535 (0.368-0.701)	57.9 (39.5-76.2)	98.3 (97.0-99.7)	64.0 (62.6-65.2)

	DeepSeek-R-Distill-Qwen	32B	0.765 (0.682-0.848)	0.452 (0.285-0.619)	55.5 (38.2-72.8)	97.5 (96.1-98.9)	51.9 (50.5-53.4)
	Qwen2.5	32B	0.758 (0.667-0.848)	0.467 (0.291-0.643)	53.3 (34.6-72.1)	98.2 (97.0-99.5)	57.0 (55.6-58.2)
	MedGemma3	27B	0.779 (0.687-0.871)	0.508 (0.317-0.699)	58.2 (39.8-76.7)	97.6 (95.3-99.9)	52.6 (50.9-54.3)
	Baichuan-M1	14B	0.783 (0.701-0.865)	0.530 (0.363-0.697)	57.8 (41.1-74.5)	98.8 (97.9-99.6)	54.7 (53.3-56.0)
	WinGPT2-Gemma2	9B	0.705 (0.638-0.772)	0.364 (0.207-0.521)	44.3 (29.9-58.6)	96.7 (94.3-99.1)	31.6 (30.1-32.7)
	Llama3	8B	0.678 (0.604-0.752)	0.324 (0.154-0.493)	39.7 (24.6-54.8)	95.9 (93.0-98.8)	30.4 (29.2-31.8)
Structured findings + clinical information	Deepseek-R1	671B	0.944 (0.921-0.966)	0.837 (0.779-0.894)	89.6 (84.8-94.3)	99.2 (98.8-99.6)	87.1 (85.9-88.0)
	Qwen3	235B	0.920 (0.889-0.951)	0.606 (0.478-0.733)	87.8 (81.4-94.1)	96.2 (95.4-97.0)	79.6 (78.6-80.6)
	GPT-OSS	120B	0.867 (0.808-0.926)	0.667 (0.550-0.783)	74.7 (62.7-86.8)	98.7 (98.0-99.4)	70.1 (68.7-71.3)
	Llama3	70B	0.854 (0.789-0.920)	0.675 (0.551-0.799)	72.1 (58.7-85.5)	98.8 (98.0-99.6)	69.9 (68.8-71.3)
	DeepSeek-R-Distill-Qwen	32B	0.805 (0.742-0.868)	0.523 (0.380-0.666)	63.4 (50.3-76.4)	97.6 (96.2-99.0)	54.2 (52.7-55.5)
	Qwen2.5	32B	0.814 (0.743-0.886)	0.577 (0.435-0.719)	64.5 (49.7-79.2)	98.4 (97.3-99.5)	60.7 (59.5-62.1)
	MedGemma3	27B	0.825 (0.751-0.899)	0.591 (0.433-0.749)	67.0 (52.3-81.7)	98.0 (95.6-100.3)	56.2 (54.6-57.5)

Baichuan-M1	14B	0.817<br>(0.753-0.881)	0.599<br>(0.461-0.737)	64.6<br>(51.6-77.5)	98.9<br>(98.2-99.6)	57.8<br>(56.2-59.3)
WinGPT2-Gemma2	9B	0.728<br>(0.666-0.790)	0.401<br>(0.260-0.543)	48.8<br>(35.5-62.1)	96.8<br>(94.4-99.3)	30.8<br>(29.5-32.2)
Llama3	8B	0.714<br>(0.643-0.785)	0.365<br>(0.203-0.528)	46.6<br>(32.3-60.9)	96.2<br>(94.0-98.4)	31.5<br>(30.3-32.6)

Data in parentheses are 95% CIs. AUROC: Area Under the Receiver Operating Characteristic Curve; AUPRC: Area Under the Precision-Recall Curve.

Table S3. The detailed overall performance metrics under Top-1, Top-2, and Top-3 evaluation settings.

Groups	Metrics	Top-1	Top-2	Top-3
**Straightforward cases**	AUROC	0.967	0.994	0.997
	AUPRC	0.923	0.981	0.989
	Sensitivity(%)	94.1	99.0	99.4
	Specificity(%)	99.4	99.7	99.9
	Accuracy(%)	92.0	97.3	98.3
	Mean Reciprocal Rank	0.982	0.988	0.988
**Challenging cases**	AUROC	0.932	0.985	0.993
	AUPRC	0.741	0.902	0.956
	Sensitivity(%)	87.8	97.5	98.8
	Specificity(%)	98.5	99.6	99.8
	Accuracy(%)	80.0	93.1	96.5
	Mean Reciprocal Rank	0.926	0.952	0.956
**Total cases**	AUROC	0.944	0.988	0.994
	AUPRC	0.837	0.955	0.978
	Sensitivity(%)	89.6	97.8	99.0
	Specificity(%)	99.2	99.7	99.9
	Accuracy(%)	87.2	95.7	97.6
	Mean Reciprocal Rank	0.960	0.973	0.975

AUROC: Area Under the Receiver Operating Characteristic Curve; AUPRC: Area Under the Precision-Recall Curve. Accuracy values reflect patient-level complete-match. For multi-label cases, a case was scored as correct only if all reference diagnostic labels were covered, with each label appearing in the distinct top-k prediction list (k = 1-3).

Table S4. The diagnostic performance of the individual radiologist with and without AI support.

Group	AUROC w.o. /w. AI	*P* value	AUPRC w.o. /w. AI	*P* value	Sensitivity(%) w.o. /w. AI	*P* value	Specificity(%) w.o. /w. AI	*P* value	Accuracy (%) w.o. /w. AI	*P* value	Reading time (s)	*P* value
Junior R1	0.894 / 0.963	0.002	0.735 / 0.878	<0.001	80.7 / 93.2	0.006	98.1 / 99.3	0.021	70.8 / 89.6	<0.001	71/60	<0.001
Junior R2	0.892 / 0.964	0.002	0.730 / 0.880	<0.001	80.3 / 93.5	0.005	98.0 / 99.3	0.018	70.0 / 89.6	<0.001	67/59	<0.001
Junior R3	0.890 / 0.957	0.005	0.726 / 0.859	<0.001	80.0 / 92.1	0.012	98.0 / 99.3	0.024	69.4 / 88.2	<0.001	68/59	<0.001
Senior R4	0.926 / 0.975	0.001	0.813 / 0.915	<0.001	86.6 / 95.5	0.003	98.6 / 99.5	0.019	78.4 / 91.8	<0.001	59/49	<0.001
Senior R5	0.928 / 0.975	<0.001	0.816 / 0.911	<0.001	86.9 / 95.5	0.003	98.6 / 99.5	0.029	79.0 / 91.8	<0.001	54/48	<0.001
Senior R6	0.931 / 0.973	0.002	0.823 / 0.906	<0.001	87.5 / 95.1	0.005	98.7 / 99.5	0.037	80.2 / 91.6	<0.001	56/48	<0.001

Sensitivity and specificity were compared using t-test. The AUROC was compared using the DeLong test. Differences in the AUPRC were assessed via bootstrap-based comparison. Patient-level accuracy was evaluated using the McNemar test.Reading time was compared using the linear mixed models.
AUROC: Area Under the Receiver Operating Characteristic Curve; AUPRC: Area Under the Precision-Recall Curve.

Table S5. The detailed information on the LLMs used in this study

Model	Model size (Active size)	Developer	Architecture	Hyperparameters (Temperature/ TopP/format)	Type	Licence	Language support	Version date	Reference
Deepseek-R1	671B (37B)	DeepSeek-AI	MoE 1 shared + 256 experts Top-8	0.6/0.95/fp8	General	MIT License	Chinese, English	2025.5	https://arxiv.org/abs/2501.12948
Qwen3	235B (22B)	Alibaba	MoE 128 Experts Top-8	0.7/0.8/bf16	General	Apache License 2.0	Chinese, English	2025.7	https://arxiv.org/abs/2505.09388
GPT-OSS	120B (5.13B)	OpenAI	MoE 128 Experts Top-4	0.7/0.9/mxfp4	General	Apache License 2.0	Chinese, English	2025.8	https://cdn.openai.com/pdf/419b6906-9da6-406c-a19d-1bb078ac7637/oai_gpt-oss_model_card.pdf
Llama3.3	70B	Meta	Dense	0.7/0.9/bf16	General	Llama 3.3 Community License	Chinese, English	2024.12	https://huggingface.co/meta-llama/Llama-3.3-70B-Instruct
DeepSeek-R1-Distill-Qw	32B	DeepSeek-AI	Dense	0.6/0.95/bf16	General	MIT License	Chinese, English	2025.2	GPT-OSS-120B https://arxiv.org/abs/2501.12948

en									
Qwen2.5	32B	Alibaba	Dense	0.7/0.9/bf16	General	Apache License 2.0	Chinese, English	2024.9	https://arxiv.org/abs/2412.15115
MedGemma 3	27B	Google	Dense	0.7/0.9/bf16	Medical	health-ai-developer-foundations	Chinese, English	2025.7	https://arxiv.org/abs/2507.05201
Baichuan-M1	14B	Baichuan-Inc	Dense	0.7/0.9/bf16	Medical	Apache License 2.0	Chinese, English	2025.2	https://arxiv.org/abs/2502.12671
WinGPT2-Gemma2	9B	WinningHealth	Dense	0.7/0.9/bf16	Medical	Apache License 2.0	Chinese, English	2024.8	https://huggingface.co/winninghealth/WiNGPT2-Gemma-2-9B-Chat
Llama3.1	8B	Meta	Dense	0.7/0.9/bf16	General	Llama 3.1 Community License	Chinese, English	2024.7	https://arxiv.org/abs/2407.21783
Kimi-K2	1.04T (32B)	Moonshot	MoE 1 shared + 384 Experts Top-8	0.6/0.9/INT4	General	MIT License	Chinese, English	2025.9	https://github.com/MoonshotAI/Kimi-K2/blob/main/tech_report.pdf

Table S6. Model-side wall-clock time per case with DeepSeek-V3 as the findings-structuring model and DeepSeek-R1 as the impression-generation model.

Type of LLM inference	Subgroups	Top1 vs Top3	(a) Findings structuring (s)	(b) LLM generation (s)	Model-side total (s)
Free-text	All	Top1	0.00 ± 0.00	16.34 ± 3.66	16.34 ± 3.66
Structured+clinical	All	Top1	3.57 ± 1.33	15.95 ± 4.02	19.52 ± 4.23
Structured+clinical	Straightforward	Top1	3.23 ± 1.19	14.51 ± 3.46	17.74 ± 3.66
Structured+clinical	Challenging	Top1	4.10 ± 1.36	18.15 ± 3.81	22.25 ± 4.05
Structured+clinical	Non-contrast	Top1	3.47 ± 1.29	15.65 ± 3.93	19.12 ± 4.14
Structured+clinical	Contrast-enhanced	Top1	4.15 ± 1.39	17.70 ± 4.08	21.86 ± 4.31
Structured+clinical	All	Top3	3.57 ± 1.33	18.90 ± 5.09	22.47 ± 5.26
Structured+clinical	Straightforward	Top3	3.23 ± 1.19	18.41 ± 4.91	21.64 ± 5.05
Structured+clinical	Challenging	Top3	4.10 ± 1.36	19.65 ± 5.28	23.75 ± 5.45
Structured+clinical	Non-contrast	Top3	3.47 ± 1.29	18.93 ± 5.13	22.40 ± 5.29
Structured+clinical	Contrast-enhanced	Top3	4.15 ± 1.39	18.72 ± 4.87	22.88 ± 5.06

Times are reported as (a) structuring, (b) generation, and total.

Table S7. Representative example of the original radiology report and structured radiology report

**Original radiology report**	**Structured radiology report**						
	Anatomical location	Number	morphology	Size	Signal	Perilesional changes	Characteristic features
左侧额叶团块状异常混杂信号，T1W 低信号为主，内少许高信号，T2W、FLAIR 稍高信号，内少许低信号，累及胼胝体膝部，大小约 60mm×63mm×72mm，周围大片水肿，中线略右移，左侧侧脑室受压，增强扫描可见不均匀花环样强化，内部可见无强化坏死区。右侧基底节小片状异常信号，DWI 高信号，ADC 低信号。双侧额顶叶脑室周边白质可见多发斑点状异常信号，T1W 等低信号，T2W、FLAIR 高信号。各脑池及脑室扩大，	left frontal lobe	one	mass-like	60mm×63mm×72mm	T1WI: hypointense signal with scattered hyperintense foci.<br>T2WI: mildly hyperintense signal with areas of hypointensity.<br>Flair: mildly hyperintense signal with areas of hypointensity.	extensive perilesional edema	Genu of the corpus callosum involved.<br>Mild midline shift to the right.<br>Heterogeneous ring-enhancement with non-enhancing necrotic areas within the lesion.

脑沟增宽加深。<br>A mass-like heterogeneous signal abnormality is noted in the left frontal lobe, predominantly hypointense on T1-weighted imaging with scattered hyperintense foci. It appears mildly hyperintense on T2-weighted and FLAIR sequences with areas of hypointensity. The lesion involves the genu of the corpus callosum and measures approximately 60mm×63mm×72mm, surrounded by extensive perilesional edema. Mild midline shift to the right is observed, with compression of the left lateral ventricle. Post-contrast imaging demonstrates heterogeneous ring-enhancement with non-enhancing necrotic areas within the lesion. A small patchy abnormal signal is seen in the right basal ganglia, showing high signal on DWI and low signal on ADC mapping. Multiple punctate abnormal signals are present in the periventricular white matter of bilateral frontal and parietal lobes, appearing iso- to	right basal ganglia	one	small patchy	null	DWI: hyperintense signal<br>ADC: hypointense signal	null	null
	periventricular white matter of bilateral frontal and parietal lobes	multiple	punctate	null	T1WI: Iso- to hypointense signal .<br>T2WI: hyperintense signal.<br>Flair: hyperintense signal.	null	null
	null	null	null	null	null	null	Enlargement of cerebral cisterns and ventricles.<br>Widened and deepened cerebral sulci.

hypointense on T1-weighted images and hyperintense on T2-weighted and FLAIR sequences. Enlargement of cerebral cisterns and ventricles is noted, along with widened and deepened cerebral sulci.	

Table S8. The distribution characteristics of error types in structured brain MRI reports by DeepSeek-V3.

**Error type**	**Total cases**	**Correct cases**	**Error cases**	**Accuracy**	**Error rate**
Anatomical location	500	498	2	0.996	0.004
Number	500	500	0	1	0
Morphology	500	498	2	0.996	0.004
Signal characteristics	500	494	6	0.988	0.012
Perilesional changes	500	499	1	0.998	0.002
Other characteristic imaging features	500	500	0	1	0

Table S9. The overall performance metrics comparisons using structured findings from DeepSeek-V3 versus Kimi-K2.

Model	Metric	DeepSeek-V3	Kimi-K2	Diff	p-value	Sig
	AUROC	0.944	0.936	-0.008	0.570	
	AUPRC	0.837	0.817	-0.020	0.685	
Deepseek-R1 (671B)	Sensitivity(%)	89.6	88.2	-1.3	0.716	
	Specificity(%)	99.2	99.0	-0.2	0.716	
	Accuracy(%)	87.1	85.6	-1.6	0.021	*
	AUROC	0.920	0.922	0.002	0.733	
	AUPRC	0.606	0.655	0.049	0.621	
Qwen3 (235B)	Sensitivity(%)	87.8	87.3	-0.4	0.418	
	Specificity(%)	96.2	97.1	0.9	0.418	
	Accuracy(%)	79.6	81.8	2.1	0.012	*
	AUROC	0.867	0.858	-0.009	0.655	
	AUPRC	0.667	0.649	-0.017	0.758	
GPT-OSS (120B)	Sensitivity(%)	74.7	73.1	-1.6	0.757	
	Specificity(%)	98.7	98.5	-0.1	0.757	
	Accuracy(%)	70.1	69.7	-0.4	1.000	
	AUROC	0.854	0.833	-0.022	0.623	
	AUPRC	0.675	0.624	-0.051	0.623	
Llama3 (70B)	Sensitivity(%)	72.1	68.1	-4.0	0.678	
	Specificity(%)	98.8	98.5	-0.3	0.678	
	Accuracy(%)	69.9	67.2	-2.7	0.001	***
	AUROC	0.805	0.809	0.004	0.804	
DeepSeek-R-Distill-Qwen (32B)	AUPRC	0.523	0.562	0.39	0.158	
	Sensitivity(%)	63.4	63.9	0.5	0.867	

	Specificity(%)	97.6	97.9	0.3	0.119
	Accuracy(%)	54.2	54.3	0.1	0.849
	AUROC	0.814	0.816	0.001	0.612
	AUPRC	0.577	0.577	-0.000	0.619
Qwen2.5 (32B)	Sensitivity(%)	64.5	64.9	0.5	0.718
	Specificity(%)	98.4	98.2	-0.2	0.718
	Accuracy(%)	60.7	62.5	1.7	0.174
	AUROC	0.825	0.819	-0.005	0.761
	AUPRC	0.591	0.576	-0.015	0.764
MedGemma3 (27B)	Sensitivity(%)	67.0	66.2	-0.8	0.819
	Specificity(%)	98.0	97.7	-0.3	0.819
	Accuracy(%)	56.2	56.2	-0.1	1.000
	AUROC	0.817	0.814	-0.003	0.814
	AUPRC	0.599	0.589	-0.011	0.827
Baichuan-M1 (14B)	Sensitivity(%)	64.6	64.2	-0.3	0.880
	Specificity(%)	98.9	98.6	-0.2	0.880
	Accuracy(%)	57.8	57.6	-0.1	1.000
	AUROC	0.728	0.709	-0.019	0.600
	AUPRC	0.401	0.378	-0.023	0.681
WinGPT2-Gemma2 (9B)	Sensitivity(%)	48.8	45.3	-3.5	0.793
	Specificity(%)	96.8	96.5	-0.3	0.793
	Accuracy(%)	30.8	30.0	-0.8	1.000
	AUROC	0.714	0.703	-0.011	0.884
	AUPRC	0.365	0.372	0.006	0.841
Llama3 (8B)	Sensitivity(%)	46.6	44.2	-2.4	0.893
	Specificity(%)	96.2	96.5	0.3	0.893

Accuracy(%)	31.5	32.1	0.6	1.000

AUROC: Area Under the Receiver Operating Characteristic Curve; AUPRC: Area Under the Precision-Recall Curve.

Table S10. Generalized estimating equation (GEE) analysis of AI assistance and reader experience on diagnostic correctness.

Predictor	Coefficient (β)	Std. error	z	p-value	Odds ratio (OR)
Intercept	3.6266	0.020	177.60	<0.001	37.6
AI assistance	0.9367	0.018	52.08	<0.001	2.55
Experience	0.2003	0.018	11.18	<0.001	1.22
AI & Experience	−0.0607	0.016	−3.72	<0.001	0.94

The dependent variable was case-level correctness (0/1) across 96,000 reader–case–condition observations. A binomial GEE with logit link and exchangeable working correlation structure was fitted, clustering on reader (6 clusters):

$$Performance \sim \beta_0 \cdot AI_{assistence} + \beta_1 \cdot Experience + \beta_2 \cdot (AI_{assistence} \& Experience)$$

Table S11. Disease-level macro performance of DeepSeek-R1 under four input settings and number of diseases with FDR-significant changes versus the baseline.

Metric (disease-level)	Free-text w/o clinical info	Free-text w clinical info	FDR-significant diseases vs baseline	Structured, w/o clinical info	FDR-significant diseases vs baseline	Structured w clinical info	FDR-significant diseases vs baseline
**AUROC**	0.854 (0.847–0.862)	0.902 (0.895–0.908)	8/16 (7 ↑, 1 ↓)	0.901 (0.892–0.909)	10/16 (10 ↑, 0 ↓)	0.944 (0.938–0.951)	14/16 (14 ↑, 0 ↓)
**AUPRC**	0.617 (0.602–0.631)	0.725 (0.705–0.740)	12/16 (11 ↑, 1 ↓)	0.732 (0.717–0.748)	12/16 (12 ↑, 0 ↓)	0.837 (0.819–0.851)	16/16 (16 ↑, 0 ↓)
**Sensitivity**	72.7 (71.3–74.2)	82.1 (80.6–83.6)	4/16 (4 ↑, 0 ↓)	81.3 (80.0–82.9)	8/16 (8 ↑, 0 ↓)	89.6 (88.3–90.7)	11/16 (11 ↑, 0 ↓)
**Specificity**	98.2 (98.1–98.3)	98.2 (98.1–98.4)	10/16 (8 ↑, 2 ↓)	98.9 (98.8–99.0)	8/16 (8 ↑, 0 ↓)	99.2 (99.1–99.3)	11/16 (10 ↑, 1 ↓)

Baseline is the "Free-text w/o clinical information" setting. For each alternative input setting, the rightmost columns report the number of diseases (out of 16) with FDR-corrected $p < 0.05$ and the direction of change (↑ improvement, ↓ deterioration) for the corresponding disease-level metric. AUROC: Area Under the Receiver Operating Characteristic Curve; AUPRC: Area Under the Precision-Recall Curve.

Table S12. Disease-level macro performance of DeepSeek-R1 under top-1 vs top-3 settings and number of diseases with FDR-significant changes versus the baseline.

Metric	Straightforward Top1	Straightforward Top3	Straightforward FDR-significant	Challenging Top1	Challenging Top3	Challenging FDR-significant	Total Top1	Total Top3	Total FDR-significant
**AUROC (disease-level)**	0.967 (0.967-0.968)	0.997 (0.996-0.997)	9/10	0.932 (0.930-0.933)	0.993 (0.993-0.993)	13/15	0.944 (0.943-0.945)	0.994 (0.994-0.994)	16/16
**AUPRC (disease-level)**	0.923 (0.922-0.924)	0.989 (0.989-0.989)	8/10	0.741 (0.738-0.743)	0.956 (0.955-0.957)	15/15	0.837 (0.836-0.838)	0.978 (0.977-0.978)	16/16
**Sensitivity (disease-level)**	96.9 (95.3-97.9)	99.6 (98.7-99.8)	7/10	92.4 (89.2-94.5)	99.0 (97.2-99.5)	10/15	95.0 (93.2-96.3)	99.3 (98.4-99.7)	14/16
**Specificity (disease-level)**	99.4 (98.9-99.6)	99.9 (99.6-100.0)	4/10	98.7 (98.1-99.2)	99.8 (99.5-99.9)	10/15	99.3 (99.0-99.5)	99.9 (99.7-99.9)	13/16
**Accuracy (patient-level)**	91.9 (90.8-92.9)	98.3 (97.7-98.7)	1/1	79.9 (78.0-81.8)	96.5 (95.5-97.2)	1/1	87.2 (86.2-88.2)	97.6 (97.1-98.0)	1/1

AUROC: Area Under the Receiver Operating Characteristic Curve; AUPRC: Area Under the Precision-Recall Curve.

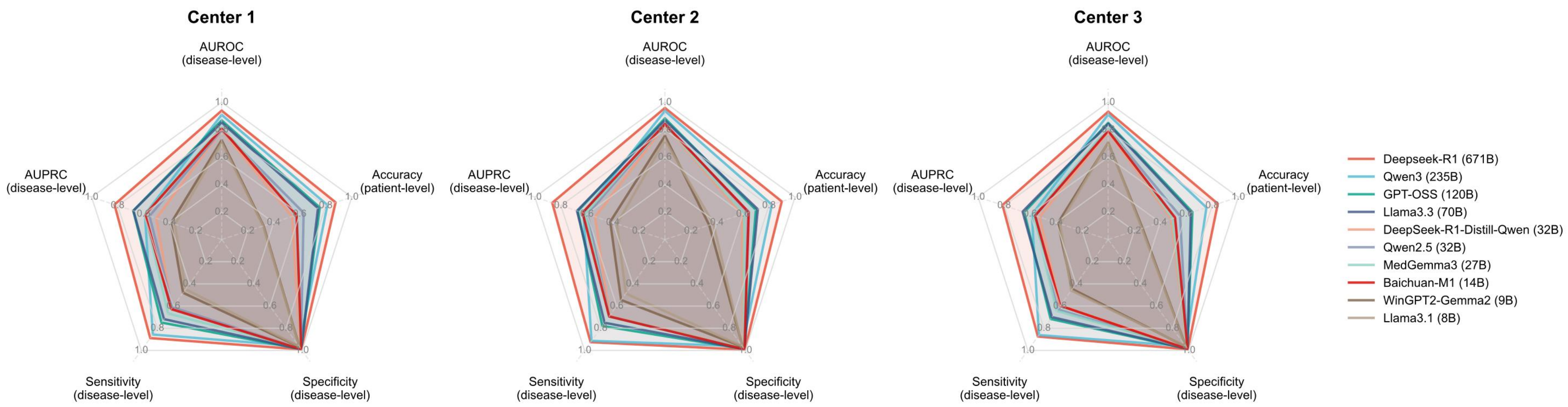

Figure. S1 Performance metrics of 10 LLMs in the separate three centers. Radar charts were constructed to illustrate the performance of 10 LLMs in **A.** Center 1; **B.** Center 2; **C.** Center 3.

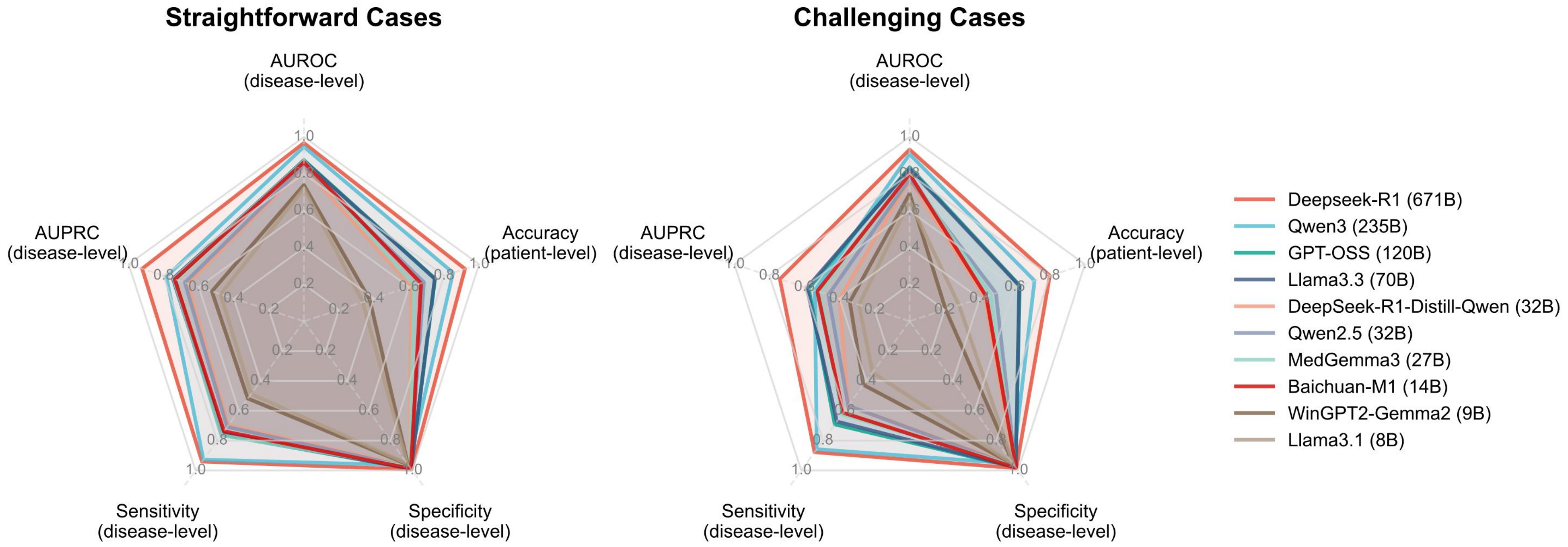

Figure. S2 Performance metrics of 10 LLMs in straightforward cases and challenging cases. Radar charts were constructed to illustrate the performance of 10 LLMs in **A.** Straightforward cases; **B.** Challenging cases.

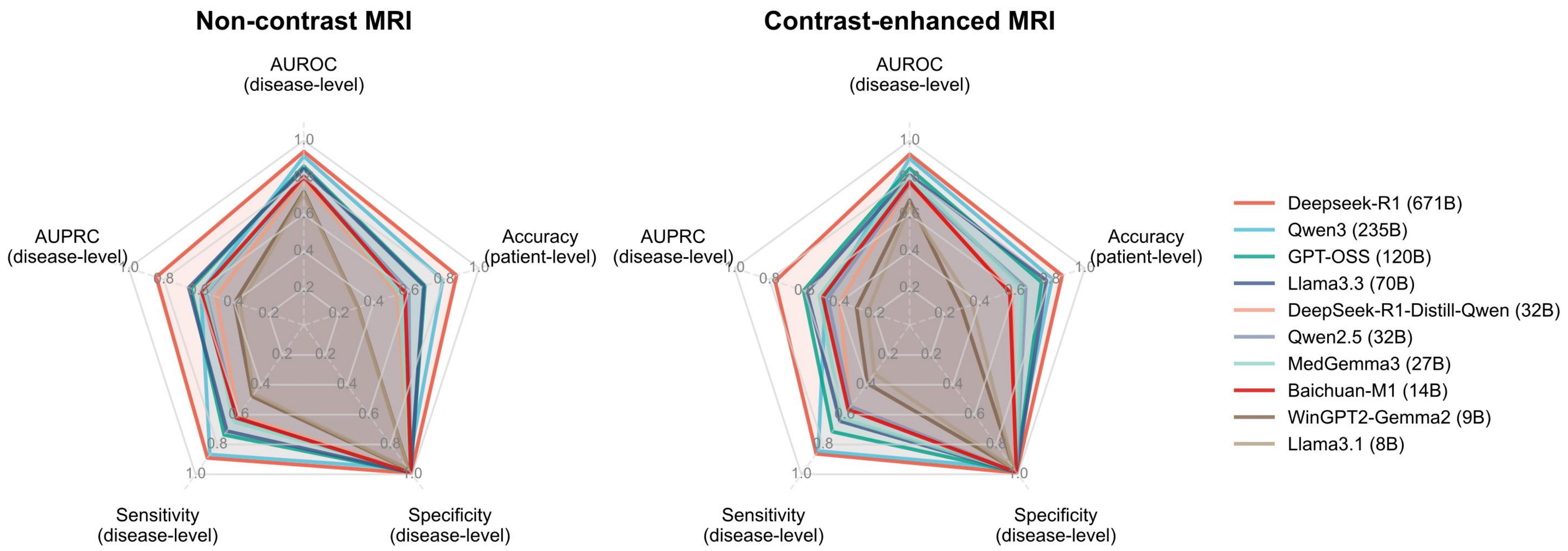

Figure. S3 Performance metrics of 10 LLMs in non-contrast MRI cases and contrast-enhanced cases. Radar charts were constructed to illustrate the performance of 10 LLMs in **A.** non-contrast MRI cases; **B.** contrast-enhanced cases.

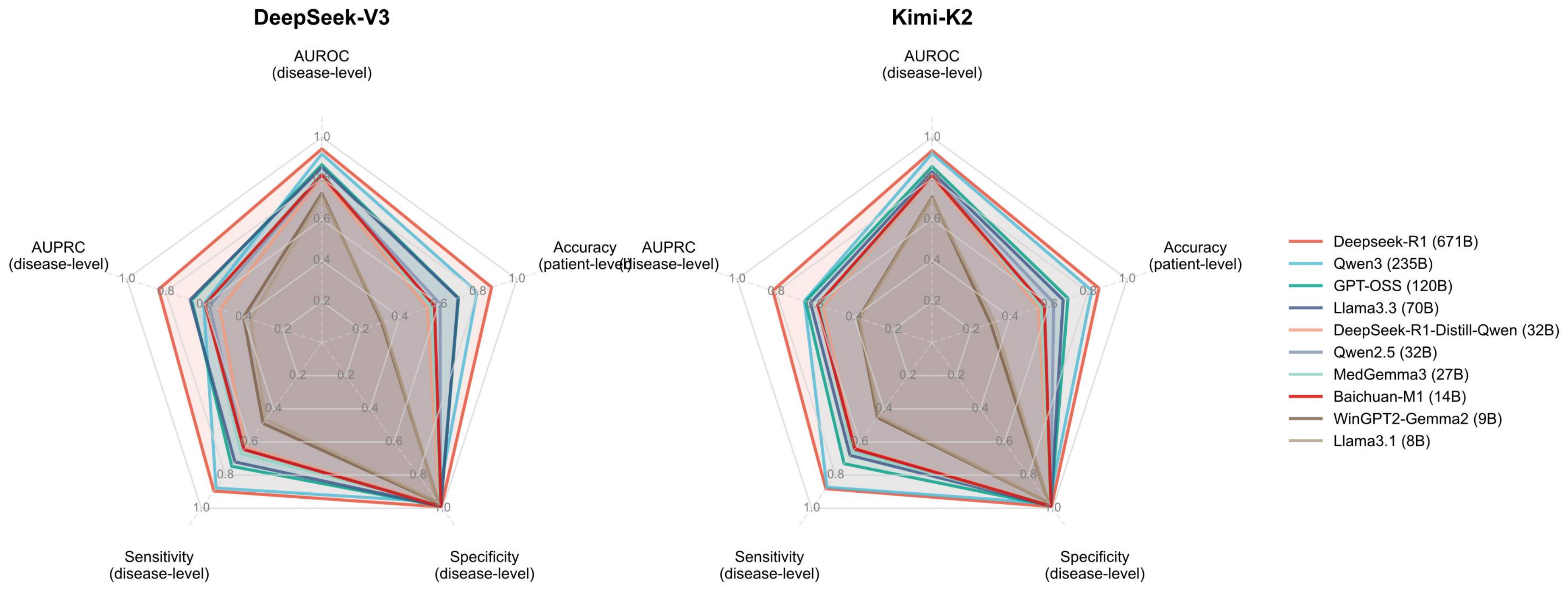

Figure. S4 Performance metrics of 10 LLMs using structured imaging findings generated by DeepSeek-V3 versus Kimi-K2. Radar charts were constructed to illustrate the performance of 10 LLMs in A. using structured imaging findings generated by DeepSeek-V3; B. using structured imaging findings generated by Kimi-K2.

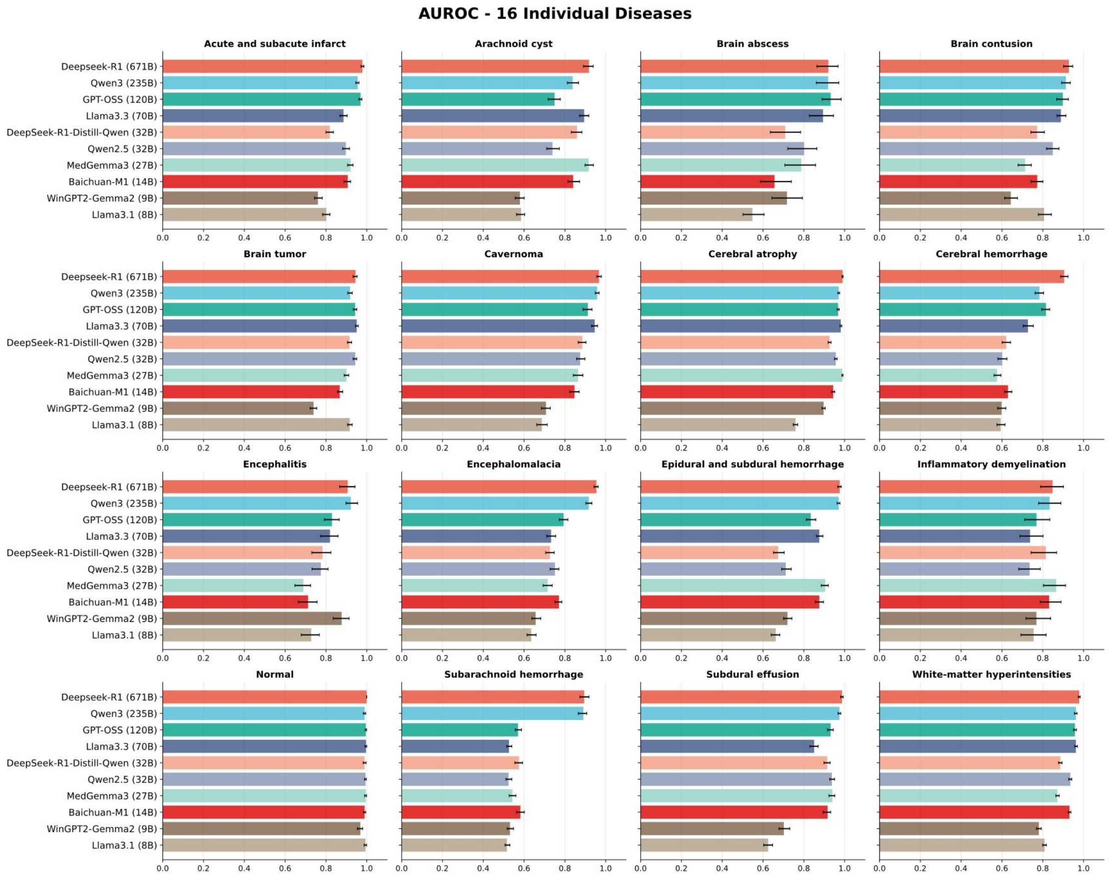

Figure. S5 AUROC metric of 10 LLMs for each individual disease category.

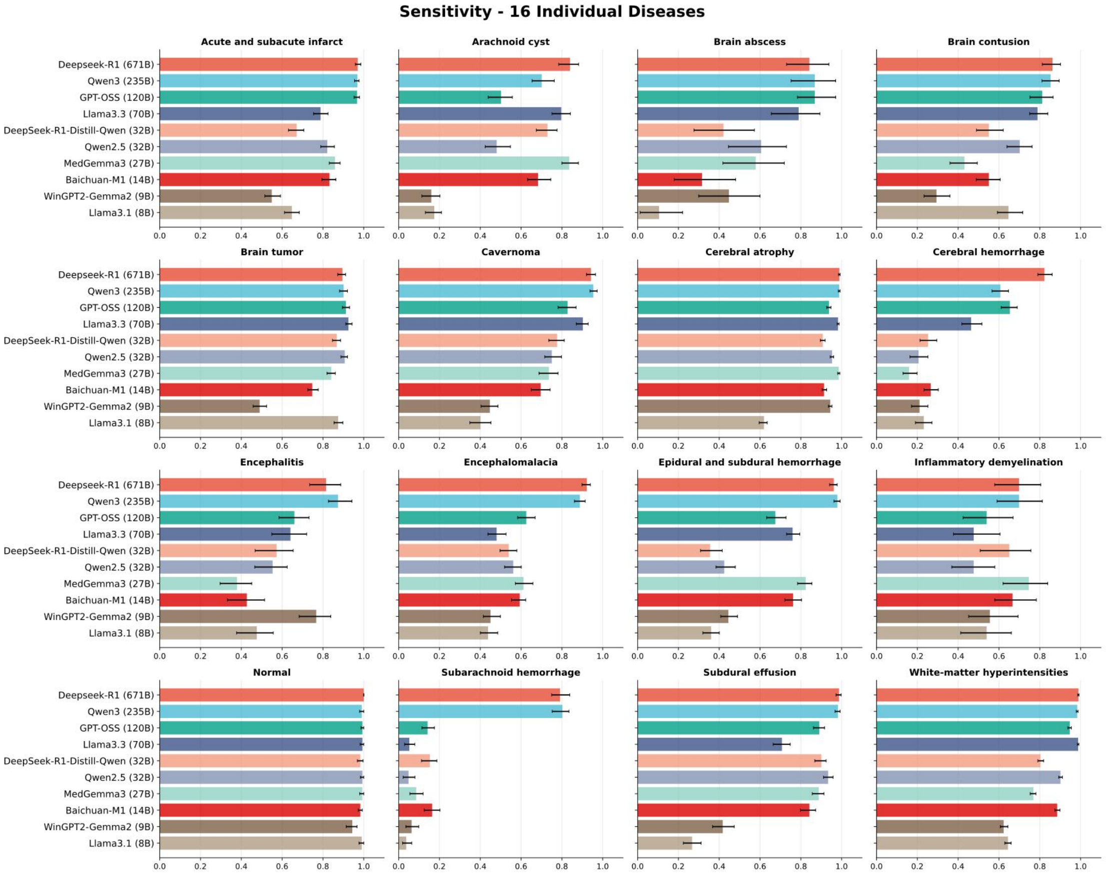

Figure. S6 Sensitivity metric of 10 LLMs for each individual disease category.

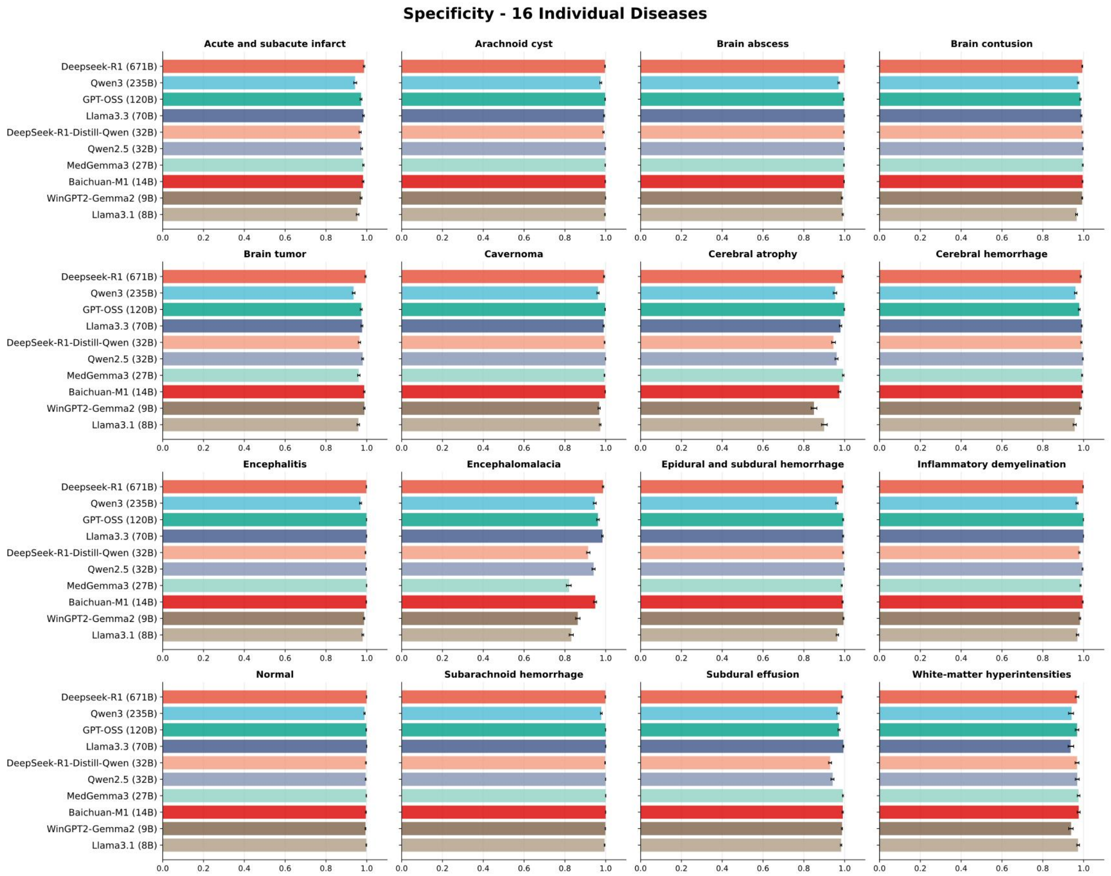

Figure. S7 Specificity metric of 10 LLMs for each individual disease category.
